# Trends in Memory Function and Memory Impairment Among Older Adults in the United States and Europe, 1996–2018

**DOI:** 10.1093/gerona/glae154

**Published:** 2024-11-07

**Authors:** Mikko Myrskylä, Jo Mhairi Hale, Daniel C Schneider, Neil K Mehta

**Affiliations:** Max Planck Institute for Demographic Research, Rostock, Germany; Helsinki Institute for Demography and Population Health, University of Helsinki, Helsinki, Finland; Max Planck Institute for Demographic Research, Rostock, Germany; School of Geography and Sustainable Development, University of St Andrews, St Andrews, UK; Max Planck Institute for Demographic Research, Rostock, Germany; Department of Epidemiology, School of Public and Population Health, University of Texas Medical Branch, Galveston, Texas, USA; (Medical Sciences Section)

**Keywords:** Comparative analysis, Dementia, Memory impairment, Trends

## Abstract

**Background:**

Single-country studies document varying time trends in memory function and impairment. Comparative analyses are limited.

**Methods:**

We used self-respondent data on adults aged 50+ years in 13 countries from 3 surveys (United States: Health and Retirement Study, 1998–2018; England: English Longitudinal Study of Ageing, 2002–2018; 11 European countries: Survey of Health, Ageing and Retirement in Europe, 2004–2019). Memory is measured with tests of immediate and delayed word recall. Unweighted age- and gender-adjusted mixed effects regression models as well as models with adjustments for additional sociodemographic characteristics and health behaviors were examined. Heterogeneity in trends by gender, age group, and educational attainment was measured.

**Results:**

The age-adjusted 10-year improvement in average test score is 0.04 standard deviations (*SD*s) (95% confidence interval [CI]: 0.03, 0.05) in the United States, 0.17 *SD*s (95% CI: 0.15, 0.19) in England, and 0.24 *SD*s (95% CI: 0.23, 0.25) in SHARE countries. Trends are largely similar across gender, age groups, and educational attainment. Regional differences in trends remain after adjustment for potential mechanisms. The difference between the United States and other countries is particularly large under 75 years of age compared to 75 years and older.

**Conclusions:**

Pace of improvement in memory function varies strongly across countries. On average, the 11 European countries studied had the fastest improvement, followed by England. The trend in the United States indicates improvement, but at a much slower pace compared to that in England and other European countries. Uncovering the causes for the cross-country heterogeneity in time trends, and in particular the reasons for the comparatively poor performance of the United States, should be both a research and public health priority.

Cognitive impairment is a key health challenge of aging populations. Globally, over 55 million individuals are estimated to have dementia; this number is expected to triple by 2050 (1). Already now, Alzheimer’s disease and related dementias (ADRD) are the leading cause of death in the United Kingdom, the sixth leading cause of death in the United States, and the seventh globally (2). Cognitive impairment, including ADRD, impacts not only the individuals directly affected but also places a substantial burden on families, caregivers, and healthcare systems.

There is no cure or efficacious treatment for Alzheimer’s disease, which is the most common cause of dementia. Thus, monitoring population-level trends in cognitive function is of major importance when anticipating the aggregate burden of cognitive impairment. Cognitive impairment is strongly age-dependent. For example, the 2019 global prevalence of dementia is estimated to be 3% at ages 70 to 74 (3%–4% in the United Kingdom, Western Europe, and United States), doubling by ages 75–79, then increasing to 25% for those aged 85+ (United Kingdom 22%, Western Europe 25%, and United States 31%) (1). A key predictor of the burden of cognitive impairment at the population level therefore is the population age structure.

However, age-specific incidence of dementia may vary over time and place. In reviews of studies conducted in Sweden, Spain, England, the Netherlands, France, the United States, Japan, and Nigeria, evidence indicates prevalence or incidence of cognitive impairment has declined over recent decades (3,4), suggesting that the impact of population aging on cognitive impairment is not deterministic, and can perhaps even be mitigated. However, other studies document opposite or stable trends (5–8). Within countries, time trends in cognitive impairment also may vary across subpopulations, for example, by race/ethnicity or level of education (8,9).

The existing evidence on the variation in time trends of cognitive impairment comes primarily from single-country studies (3,4). One exception is that Wolters and colleagues analyze data from smaller, population-based samples from several cities across the United States, England, and Europe; they find declining trends, but highlight the need for additional work on geographically and ethnically diverse samples (10). Comparative, cross-country analyses of the time trends from a large number of countries are limited. Documenting the cross-country variation, including within-population heterogeneity, is important for 2 key reasons. First, the time trends are crucial for anticipating the burden of dementia. Second, analysis of the sources of the variation may help to improve our understanding of both risk and protective factors for cognitive impairment. Identifying modifiable risk factors is a primary focus for this incurable disease (11).

We use 3 different data sources that cover a total of 13 countries to analyze time trends in one key dimension of cognitive function that is measured consistently across all 13 countries—memory function. We analyze within-country heterogeneity in time trends in subpopulations defined by gender, age, and education. To explore mechanisms, we adjust our estimates for potential protective and risk factors, including education, health and health behaviors, partnership status, and migration background. Our results are based on publicly available data sets and replication code is posted at https://osf.io/pw8zg.

## Data and Methods

### Study Population

We use the Health and Retirement Study (HRS) from the United States (12), the English Longitudinal Study of Ageing (ELSA) (13) and the Survey of Health, Ageing and Retirement in Europe (SHARE) (14). These sibling studies are all nationally representative panel surveys that collect information of residents older than 50 years on demographic factors, educational attainment, socioeconomic characteristics, and health and well-being, including memory function. We use data from the harmonized project Gateway to Global Aging Data (g2aging.org) (15); and for the HRS, we additionally use the HRS tracker file and RAND HRS longitudinal file for proxy responses, interview mode, and additional demographic information. The exact data sources and instructions on how to obtain the data can be found in the replication script.

We restrict the analysis to survey waves in which a harmonized set of memory measures were available (HRS 1996–2018, ELSA 2002–2018, SHARE 2004–2019, but not 2009). We retain only those 11 SHARE countries that cover the span from wave 1 (2004) to wave 8 (2019): Austria, Belgium, Denmark, France, Germany, Greece, Israel, Italy, Spain, Sweden, and Switzerland, and refer to this aggregate as “SHARE-11.” For easier presentation of results, we label wave years as even-numbered years (1996–2018), even though SHARE field times largely fell on odd-numbered years (2007–2019).

The starting years in the 3 surveys vary. We conduct analyses using both the maximum length of the data (starting years 1996 for HRS, 2002 for ELSA, and 2004 for SHARE) and by restricting the data to a common starting year 2004.

### Measures

Memory function is defined based on 2 indicators from the modified Telephone Interview for Cognitive Status (TICS-M) that reflect episodic memory, an important dimension of neurophysiological health, and that are consistent across all 3 surveys and the widest time range: immediate (0–10 points) and delayed word recall (0–10 points). The range is 0–20; higher scores indicate better memory function. For the HRS, we use the University of Michigan Survey Research Center’s imputed TICS-M values (16). ELSA and SHARE do not release analogous imputation files, but we address this inconsistency in robustness checks. Previous research suggests that the word recall scores across the 3 surveys are comparable (17). One difference is the usage of 4 identical (rotating) word lists of HRS and ELSA, whereas SHARE uses different word lists. In addition, in SHARE waves 1 and 2, a single (ie, nonrotating) word list is used. In SHARE wave 3, no cognitive interviews took place, and starting in SHARE wave 4 word lists are rotated. The nonrotating word list of the first 2 SHARE waves is likely to increase practice effects, whereas the 4-year spacing between interviews of SHARE waves 2 and 4 is likely to decrease them, so these effects should be partially offsetting. We include a practice effects measure in the regressions to account for any remaining net effects (see below).

We model memory in 2 ways. The first approach is to model memory linearly based on the actual score (0–20) and the second is based on a dichotomous indicator of memory impairment. We consider the first approach based on continuous score to be the preferred approach as it avoids dichotomizing the outcome, thereby losing statistical power, and also because of the inherent difficulty of defining the threshold for impairment. With respect to the latter, there are no standard cut-points to indicate memory impairment that would be applicable to all 3 data sets. The validated cut-points for mild cognitive impairment (MCI) and dementia in the HRS are based on a broader set of cognition measures than our harmonized 20-point score (18). For the purposes of this study, we define memory impairment as the score being 1.5 or more standard deviations (*SD*) below the average score of the country-specific population aged 50–69 calculated over all waves of the samples.

The threshold of 1.5 *SD* is consistent with advice on cognitive impairment from the International Working Group on MCI (19) and produces a prevalence of memory impairment in the HRS that is close to that of the MCI prevalence previously validated using the Aging, Demographics, and Memory Study (ADAMS) (20), which uses comprehensive neuropsychological examinations to provide a diagnosis of cognitive impairment on a subset of the HRS sample (18). Although the underlying memory score represented by the 1.5 *SD* threshold will vary across countries, our focus is on comparing country-specific time trends versus comparing countries’ prevalences. We also present results based on alternative thresholds (1.3 *SD*, 1.7 *SD*, and 2.0 *SD*).

In supplementary analyses for the United States, we use a measure from proxy interviews to indicate categories of impairment when respondents do not complete the TICS-M. SHARE and ELSA (initially only about 2%–3% proxy compared to 12% in the HRS (21)) do not have analogous measures.

We control for age at interview (quadratic continuous specification), self-reported gender (woman/man), educational attainment (less than upper secondary education, upper secondary education or vocational training, or tertiary education), migration background (binary, yes for being born in another country), and partnership status (partnered/not). About 10% of respondents in ELSA do not have their education level recorded. These subjects are excluded from the analysis.

We use 8 health measures based on whether the respondent has ever been diagnosed with a certain condition. Four of these (diabetes, high blood pressure, heart problems, and stroke) are related to the cardiovascular system, which is associated with cognitive health (22); the others are arthritis, cancer, lung disease, and psychological problems (a term summarizing survey question wording that indicates self-report of emotional, nervous, or psychiatric problems). We control for body mass index (BMI, kg/m^2^ categorized into <18.5, 18.5–24.9, 25–29.9, 30 or above), smoking (never smoker, former smoker, current smoker), and physical exercise (exercise more than once per week or not). We include a binary indicator for first memory function test in order to mitigate panel conditioning effects (23).

Due to missingness related to questions not being asked in all SHARE waves, the following carry-forward or carry-backward operations were applied: Ever diagnosed with psychological problems was carried backward from wave 2 to wave 1; exercise frequency was carried forward from wave 6 to wave 7; and about half of the observations for smoking behavior of waves 5 and 6 were carried forward to waves 6–8.

### Statistical Models

For the continuous average score, we use a linear model whose time trend coefficients are standardized by the standard deviation of the dependent variable. We employ 4 different model specifications (M1–M4), where each model is fit separately for country-specific data and for SHARE-11. M1 regresses memory score on (continuous) calendar year and includes controls for age, gender, and indicator for first test. Additionally, for the United States, due to sampling specifics of the HRS, we include race/ethnicity (categorized as: non-Hispanic White, Black, non-Black Hispanic, and “other”), interview mode (telephone vs face-to-face), and the interaction between interview mode and gender. M1 is our key model that documents the time trends.

In M2, we add education to M1 to study whether the time trends are driven by changing educational composition. M3 adds health behaviors, underlying health conditions, partnership status, and migration background to M1. This allows us to analyze whether the time trends are driven by changes in the prevalence of these risk factors. Finally, M4 comprises the full set of controls.

To explore within-country heterogeneity, we estimate model M1 by gender, age (50–64, 65–74, 75+), and education, separately for each country and also for SHARE-11. For the binary measure of memory impairment, we use logistic regression and 4 models M1–M4 with the same covariate specifications as for continuous score.

All regressions (linear or logistic) are estimated as mixed effects models that feature a random intercept as well as a random (age) slope. The inclusion of a random slope is motivated by a comparison of AIC and BIC model selection criteria. Mixed models provide valid inference in the presence of within-subject correlation of error terms. Additionally, mixed effects models have desirable properties with respect to missing data mechanisms, which is, with respect to (differential) nonresponse, attrition, or mortality, a concern for the present research question, because all 3 of these mechanisms are potentially related to cognition. Our model choice is in part inspired by 2 papers by Stolz and colleagues (24,25) who examine age profiles of frailty, encountering similar missing data problems. In (25), they use mixed effects models, robust to missing-at-random (MAR) missingness (26), and joint models, which are robust to observations not-missing-at-random (NMAR), and detect very little difference between them. In a similar fashion, our main model specification is a mixed-effects one, with joint modeling pursued in the robustness section. Our mixed effects model leaves the covariance between the 2 random effects unrestricted. The estimation method is maximum likelihood. All analyses are performed in Stata 18 (27). Joint model estimates were obtained using the “stjm” package (28) for Stata. Because ELSA and SHARE do not provide longitudinal weights suitable for our analysis, all regressions are unweighted.

## Results

### Descriptives

Table 1 depicts descriptive statistics for the entire sample and for selected subperiods in order to document the evolution of descriptive numbers over time. Our sample consists of 36 886 individuals for the HRS, 68 033 for SHARE, and 11 124 for ELSA. Mean age is 67 years in each of the data sets and increasing across waves, and 54%–59% are women. Memory function score (range 0–20) is 10.0 in the 1996–1998 wave pair, 9.7 in the 2004–2006 wave pair, and 9.8 in the last 2016–2018 wave pair for the United States. For ELSA, the numbers for the 2002, 2004–2006, and 2016–2018 time spans are 9.9, 9.9, and 10.6, and for SHARE-11 wave pairs 2004–2006 and 2016–2018, they are 8.4 and 9.2, suggesting stronger improvement in SHARE-11 and ELSA than in the United States. For memory impairment, all 3 study populations exhibit declining unadjusted trends.

**Table 1.** Descriptive Characteristics of the Analytical Sample

	HRS				ELSA				SHARE		
	1996–1998	2004–2006	2016–2018	All Years	2002	2004–2006	2016–2018	All Years	2004–2006	2016–2018	All Years
Age (years, mean)	66	68	67	67	64	66	71	67	65	70	67
Women (%)	59	59	58	59	53	54	55	54	55	56	55
Memory function score (mean; range 0–20)	10.0	9.7	9.8	9.8	9.9	10.2	10.6	10.4	8.4	9.2	9.1
Memory impairment (1.5 *SD*)	13.2	12.5	11.1	12.3	16.4	15.0	14.3	14.3	16.8	13.5	14.4
Number of prior tests (mean)	1.4	4.4	6.4	4.5	1.0	2.4	7.0	4.0	1.4	4.2	2.6
Educational attainment											
Less than upper secondary	28	21	17	21	41	38	26	33	49	38	42
Upper secondary/vocational training	55	58	59	58	45	47	53	49	31	36	35
Tertiary education	17	21	24	21	14	15	21	18	20	26	24
Obesity											
Underweight (BMI <18.5)	2	1	1	1	1	1	1	1	1	1	1
Normal (BMI ≥18.5, <25)	37	34	27	32	27	27	27	27	38	39	39
Overweight (BMI ≥25, <30)	40	40	38	40	43	43	43	42	43	42	42
Obese (BMI ≥30)	21	25	34	27	30	30	30	30	18	19	18
Smoking											
Never	41	43	46	43	36	37	37	37	54	55	54
Former	42	43	40	42	47	48	54	50	28	30	29
Current	17	14	14	15	17	15	9	13	18	15	17
Ever diagnosed health conditions											
Diabetes	13	18	28	20	6	8	13	10	11	17	14
High blood pressure	43	53	62	54	35	41	47	42	37	55	45
Heart problems	20	23	24	23	14	17	26	19	14	20	16
Stroke	6	8	9	8	3	4	6	4	4	8	6
Arthritis	48	56	58	55	29	34	44	37	24	45	35
Cancer	10	13	15	13	5	7	15	10	6	13	9
Lung disease	7	8	11	9	5	6	8	6	6	11	9
Psychological problems	9	14	21	15	7	8	12	10	9	17	13
Partnered	67	64	59	63	72	70	68	70	75	73	75
Exercises ≥1 per week	44	54	49	48	61	62	62	62	70	68	69
Born in foreign country	9	10	16	12	6	6	9	8	11	9	10
Person-waves	33 470	33 894	33 212	203 400	7 239	14 309	11 712	64 659	43 871	55 477	212 776

*Note*: HRS, 1996–2018 (number of persons 36 886); ELSA, 2002–2018 (number of persons 11 124); SHARE, 2004–2018 (number of persons 68 033). Values tabulated are percentages if not otherwise noted. For each data set, averages across the first 2 waves (ELSA: first wave only) and averages across the last 2 waves as well as the total (averages across all waves) are shown; and, in addition, the average across 2004 and 2006, which are available for each data set. The waves not shown in Table 1 were included in the analysis and are shown in Supplementary Appendix Table 1A–C.

Level of education increases across all 3 data sets. The HRS population is less often partnered and reports exercising less than the ELSA and SHARE populations. Prevalence of obesity increases in HRS and SHARE, but the increase is more pronounced in HRS. The fraction of current smokers declines in each of the data sets, but this decline is slower in HRS and SHARE than in ELSA. Diabetes, high blood pressure, heart problems, and stroke, which are all risk factors for cognitive impairment, are either at a higher level in HRS or their prevalence increases faster than in ELSA and SHARE (with the exception of heart problems, which are higher in ELSA). For most other health conditions, the prevalence is highest in the HRS. These differences highlight the importance of taking these risk factors into consideration in a comparative analysis of trends in memory impairment.

### Time Trends in Average Memory Function

Table 2 shows time trend coefficients for average score based on models M1, M2, M3, and M4, using the continuous time specification. The coefficients are scaled to represent standard deviation change in average score per 10-year change in time. Supplementary Appendix Table 2A–C document the full model results, demonstrating that the coefficients for the control variables are largely in the expected direction.

**Table 2.** Linear Time Trend Coefficients for Continuous Score, Models M1–M4

			M1—Descriptive		M2—Education		M3—Health, Demographic		M4—Full Adjustment	
		*N*	*b*	95% CI	*b*	95% CI	*b*	95% CI	*b*	95% CI
Survey	HRS	203 400	0.02	[0.01, 0.02]	−0.04	[−0.04, −0.03]	0.04	[0.04, 0.05]	−0.01	[−0.02, −0.01]
	ELSA	64 658	0.18	[0.16, 0.20]	0.09	[0.07, 0.11]	0.20	[0.18, 0.22]	0.11	[0.09, 0.13]
	SHARE	212 774	0.24	[0.23, 0.25]	0.17	[0.16, 0.18]	0.26	[0.25, 0.27]	0.19	[0.18, 0.20]
	HRS 2004-	137 548	0.04	[0.03, 0.05]	−0.01	[−0.02, 0.00]	0.07	[0.06, 0.08]	0.02	[0.01, 0.03]
	ELSA 2004-	57 419	0.17	[0.15, 0.19]	0.08	[0.06, 0.10]	0.19	[0.17, 0.21]	0.10	[0.08, 0.12]
SHARE Countries	Austria	17 617	0.17	[0.13, 0.21]	0.13	[0.08, 0.17]	0.18	[0.13, 0.22]	0.13	[0.09, 0.17]
	Belgium	27 578	0.32	[0.30, 0.35]	0.26	[0.24, 0.29]	0.36	[0.33, 0.39]	0.29	[0.26, 0.32]
	Denmark	18 316	0.09	[0.05, 0.12]	0.04	[0.01, 0.08]	0.10	[0.07, 0.14]	0.06	[0.03, 0.09]
	France	22 964	0.36	[0.33, 0.39]	0.29	[0.26, 0.32]	0.37	[0.34, 0.40]	0.30	[0.27, 0.33]
	Germany	21 454	0.21	[0.17, 0.24]	0.18	[0.14, 0.21]	0.24	[0.20, 0.27]	0.20	[0.17, 0.23]
	Greece	14 313	0.27	[0.24, 0.30]	0.23	[0.20, 0.26]	0.30	[0.27, 0.33]	0.26	[0.23, 0.29]
	Israel	9 531	0.42	[0.37, 0.48]	0.34	[0.29, 0.40]	0.45	[0.40, 0.50]	0.36	[0.31, 0.42]
	Italy	22 945	0.27	[0.24, 0.30]	0.21	[0.18, 0.24]	0.30	[0.27, 0.33]	0.25	[0.22, 0.28]
	Spain	23 306	0.36	[0.33, 0.40]	0.31	[0.28, 0.34]	0.39	[0.36, 0.42]	0.34	[0.31, 0.37]
	Sweden	19 547	0.12	[0.09, 0.15]	0.05	[0.02, 0.08]	0.13	[0.09, 0.16]	0.06	[0.03, 0.09]
	Switzerland	15 203	0.30	[0.26, 0.34]	0.23	[0.19, 0.27]	0.34	[0.29, 0.38]	0.27	[0.23, 0.31]

*Note*: Based on survey/country-specific linear random effects (intercept, age slope) regressions on quasi-continuous memory score 0–20. M1 regresses memory score on calendar year and includes controls for age, gender, number of tests taken, and for the United States, race/ethnicity, and interview mode. M2 adds education to M1. M3 adds demographic variables (migration, partnership), behaviors, and underlying health conditions to M1. M4 adds education to model M3. Coefficients shown are for linear time (unit: 10 years), standardized by dividing through the standard deviation of the dependent variable.

Table 2, model M1, shows that across all countries, the time trend in average memory score is positive, but varies in magnitude. The United States has a slightly positive time trend: 0.02 (95% CI: 0.01, 0.02) with full data, 0.04 (95% CI: 0.03, 0.05) when starting in 2004. Across the SHARE countries, the time trend coefficient is 0.24 (95% CI: 0.23, 0.25). Israel (0.42), Spain (0.36), and France (0.36) have the most positive trend, and Sweden (0.12) and Denmark (0.09) the least. ELSA is slightly below the SHARE average: 0.18 (95% CI: 0.16, 0.20) with full data and 0.17 (95% CI: 0.15, 0.19) when starting in 2004.

**Figure 1.**
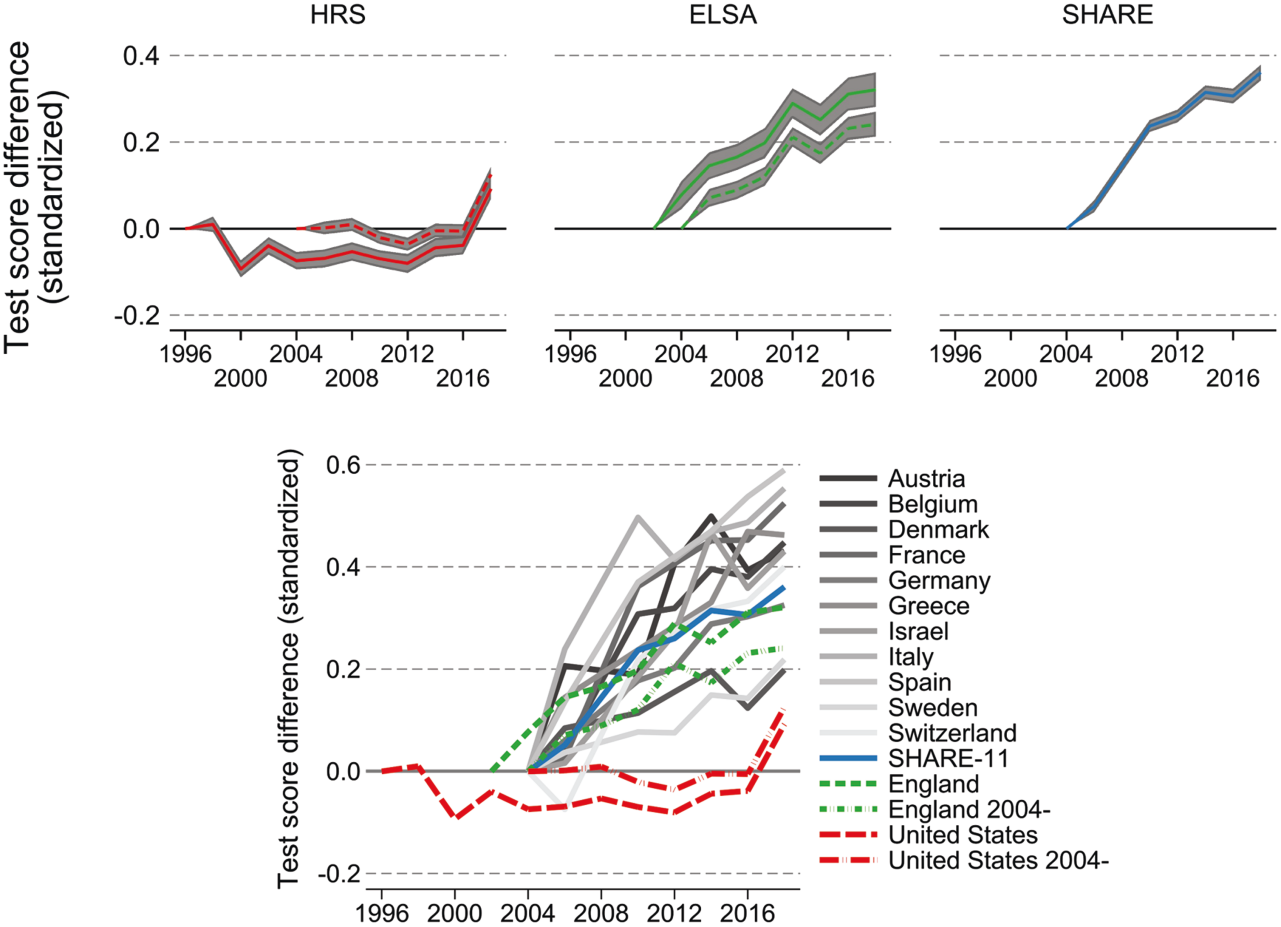
Change in continuous score over survey waves (model M1). Based on survey/country-specific linear random effects (intercept, age slope) regressions on quasi-continuous memory score 0–20. Each line depicts regression coefficient values of survey wave year indicators, standardized by dividing through the standard deviation of the dependent variable, so values are the difference in terms of standard deviations of memory score relative to the first survey year in the sample (HRS: 1996; ELSA: 2002; SHARE: 2004). The underlying model M1 adjusts for age, gender, number of tests taken, and for the United States, race/ethnicity, and interview mode.

Figure 1 illustrates these time trends based on descriptive model M1 and using wave indicators as the time specification. This figure shows, in addition to the overall time trend, shorter-term changes. The top panels show the patterns for United States, England, and SHARE-11 with associated confidence intervals, and the bottom panel shows all countries together but without confidence intervals to avoid cluttering. The overall regional patterns of robust improvement in England and SHARE countries, and more shallow improvement in the United States are also visible when the time trend is estimated by wave indicators, instead of continuous time. The figure also illustrates that this overall pattern holds both when using the full available time series or when using a shared starting year of 2004. Having a relatively flat trajectory throughout most of the observation period, the United States is clearly overall lagging behind England and SHARE countries. The most recent data points, however, suggest rapid improvement in the United States, narrowing the trend differentials to some extent.

Model 2 (Table 2) introduces controls for education. As a result, the estimated time trends become less positive (SHARE and England), or flat or negative (United States), indicating that part of the positive trends may be attributable to the expansion of education.

Model 3 introduces controls for migration background, partnership status, health behaviors, and health. On average, across the countries, the coefficients in Model 3 when compared to Model 1 are slightly more positive. This suggests that the improvement in memory scores would have been even stronger if the distribution of the risk factors had not changed. The country rankings based on Model 3, however, are similar to those in Model 1.

Model 4 jointly controls for education, migration background, partnership status, health behaviors, and health. The results of this model are also qualitatively consistent with the descriptive Model 1: The United States has a time trend that is close to flat—slightly negative −0.01 (95% CI: −0.02, −0.01) with full data, slightly positive 0.02 (95% CI: 0.01, 0.03) when starting in 2004. England and SHARE-11 have positive time trends.

**Figure 2.**
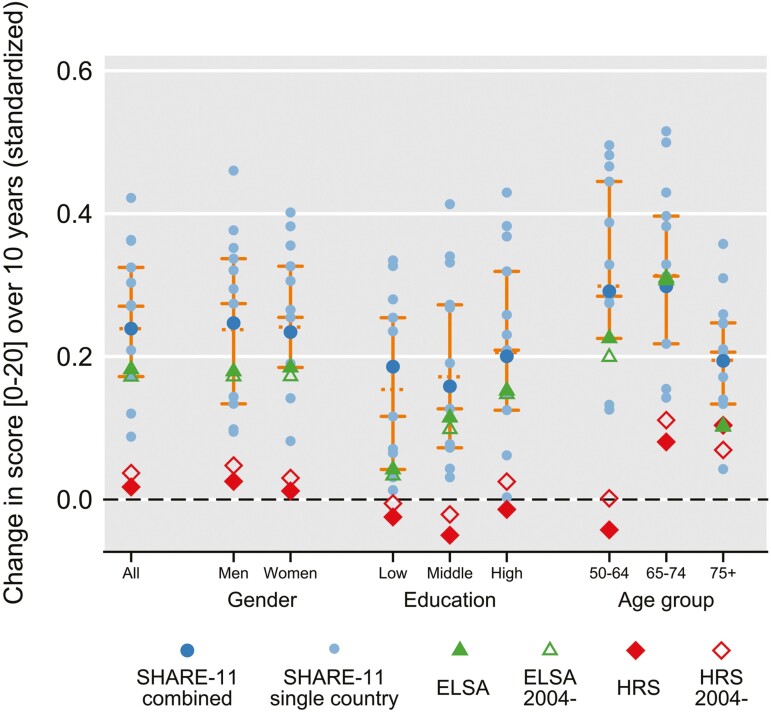
Change in continuous memory score over 10 years, by subsamples of gender, education, and age group. Based on survey/country-specific linear regressions on quasi-continuous memory score 0–20 for the full sample and for subpopulations. Samples start at the first survey year (HRS: 1996; ELSA: 2002; SHARE: 2004) unless indicated otherwise in the figure legend. Markers are regression coefficients for linear time (unit: 10 years), standardized by dividing through the standard deviation of the dependent variable, and map as follows: Light blue circles—individual SHARE countries; blue circles—combined sample for SHARE-11; green triangles—ELSA/England; red diamonds—HRS/United States. Hollow symbols are used for 2004- samples. Vertical orange lines connect 25% and 75% percentiles, calculated over all 13 countries (and so excluding the SHARE-11 total and the 2004- subsamples for the HRS and ELSA). The median and mean are also shown, using markers of orange horizontal solid and dotted lines, respectively. The underlying model M1 adjusts for age, gender, first test occasion, and for the United States, race/ethnicity, and interview mode. Values depicted along with 95% confidence intervals are tabulated in Supplementary Appendix Table 3A and B.

Figure 2 shows time trends in average memory score, based on model M1 and estimated separately by gender, age groups, and education. The results do not reveal large systematic differences in the time trajectories within these subpopulations. As in most comparisons, SHARE-11 has the steepest improvement, followed by England and then by the United States. An important exception is the pattern by age. The gap between the United States compared to SHARE-11 and England is largest in the youngest age group (50–64 years). For ages 65–74, the gap narrows somewhat, as the data suggest positive trends for the United States; however, for the other regions, the positive trend is still markedly stronger. For the oldest age group, 75 years and older, the gap further narrows. In this age group, the SHARE-11 estimate is 0.20 standard deviation improvement over a 10-year time period, and the United States and England estimates are close to each other, at roughly half of the SHARE estimate.

Table 3 shows the odds ratios for the time trend in memory impairment based on a logistic regression model (M1) and using a variety of thresholds for memory impairment. The qualitative pattern that emerges is similar to what we observe when modeling average time trend with a linear regression. In all countries, we find decreasing odds of memory impairment. The pace of decline varies, with SHARE-11 having the fastest decline, England coming second, and the United States third. This ranking holds independently of whether one uses the full data or harmonizes the starting year to 2004. Using differential thresholds (1.3 *SD*, 1.7 *SD*, or 2 *SD*) does not change the picture: SHARE countries and England on average have a faster pace of decline in memory impairment than the United States. Supplementary Appendix Figure 3 illustrates the patterns using the 1.5 *SD* threshold.

**Table 3.** Odds Ratios From Logistic Regression for 10-Year Change in Memory Score for Varying Thresholds of Impairment

			Main		Alternatives					
			1.5 *SD*		1.3 *SD*		1.7 *SD*		2.0 *SD*	
		*N*	*b*	95% CI	*b*	95% CI	*b*	95% CI	*b*	95% CI
Survey	HRS	203 400	0.86	[0.83, 0.89]	0.89	[0.86, 0.92]	0.86	[0.83, 0.89]	0.85	[0.81, 0.88]
	ELSA	64 658	0.67	[0.61, 0.73]	0.62	[0.57, 0.67]	0.72	[0.65, 0.80]	0.76	[0.67, 0.85]
	SHARE	212 774	0.51	[0.48, 0.53]	0.50	[0.47, 0.52]	0.52	[0.49, 0.56]	0.59	[0.55, 0.63]
	HRS 2004-	137 548	0.89	[0.84, 0.94]	0.90	[0.86, 0.95]	0.89	[0.84, 0.94]	0.89	[0.83, 0.95]
	ELSA 2004-	57 419	0.68	[0.61, 0.74]	0.62	[0.57, 0.67]	0.74	[0.66, 0.82]	0.77	[0.68, 0.86]
SHARE Countries	Austria	17 617			0.52	[0.42, 0.62]	0.43	[0.32, 0.54]	0.43	[0.30, 0.56]
	Belgium	27 578	0.39	[0.33, 0.45]	0.38	[0.33, 0.43]	0.44	[0.36, 0.52]	0.49	[0.38, 0.59]
	Denmark	18 316	0.87	[0.69, 1.04]	0.79	[0.66, 0.93]	0.76	[0.58, 0.95]	0.82	[0.59, 1.04]
	France	22 964	0.37	[0.30, 0.43]	0.37	[0.30, 0.43]	0.34	[0.28, 0.41]	0.36	[0.28, 0.44]
	Germany	21 454	0.69	[0.56, 0.83]	0.63	[0.53, 0.74]	0.69	[0.56, 0.83]	0.74	[0.57, 0.91]
	Greece	14 313	0.59	[0.50, 0.69]	0.56	[0.48, 0.64]	0.59	[0.50, 0.69]	0.65	[0.52, 0.78]
	Israel	9 531	0.39	[0.28, 0.50]	0.34	[0.26, 0.42]	0.39	[0.28, 0.50]	0.53	[0.35, 0.70]
	Italy	22 945	0.46	[0.38, 0.54]	0.42	[0.36, 0.48]	0.46	[0.38, 0.54]	0.52	[0.41, 0.62]
	Spain	23 306	0.41	[0.35, 0.47]	0.41	[0.35, 0.47]	0.47	[0.39, 0.55]	0.68	[0.54, 0.83]
	Sweden	19 547	0.71	[0.60, 0.81]	0.71	[0.60, 0.81]	0.73	[0.59, 0.86]	0.85	[0.67, 1.03]
	Switzerland	15 203	0.57	[0.44, 0.69]	0.45	[0.36, 0.54]	0.57	[0.42, 0.71]	0.50	[0.34, 0.66]

*Note*: Based on survey/country-specific logistic random effects (intercept, age slope) regressions on binary impairment, defined by scores that are 1.3, 1.5, 1.7, and 2 standard deviations below the mean for ages 50–69. The main results discussed in the paper use 1.5 standard deviations to define the impairment threshold. Alternative definitions employ 1.3, 1.7, and 2 standard deviations. Odds ratios shown are for linear time (unit: 10 years). The underlying model M1 adjusts for age, gender, number of tests taken, and for the United States, race/ethnicity, and interview mode.

### Robustness Checks

Below, we enumerate various robustness specifications. None of them substantially alter the main qualitative conclusions that most countries are experiencing improvements in memory function; the trend in the United States is markedly weaker than that of SHARE and England; and this holds for all subgroups except for ages 75+ in which the United States and England trends are similar, but weaker than in SHARE countries.

First, we ran additional logistic regressions based on the covariate lists of models M1–M4 in Table 2 (Supplementary Appendix Table 4). Second, there is no uniform consensus in the literature of whether and how to adjust for practice effects. We therefore modified our baseline specification to not include a practice effect measure at all (Supplementary Appendix Tables 5 and 6) or replaced the binary indicator on first test by a series of indicators capturing prior test experience (categories 0, 1, 2–3, 4–6, 7+; Supplementary Appendix Table 8). Third, for linear models, we specified age in 5-year age groups instead of continuous quadratic (Supplementary Appendix Table 7).

Further, the accumulated number of tests may vary across countries in ways that are correlated with memory. A useful robustness check would be to estimate models based only on first-time interviews in which neither selective attrition nor practice effects play a role. Unfortunately, due to differences in refresher samples, this is viable only in SHARE, where refresher samples generally capture a wide age group. In ELSA and HRS, analysis of first-timers would mean comparing different age groups at different time points. Therefore, we present the first-timer results only for SHARE (Supplementary Appendix Table 9). For the HRS, where it is possible to do so, we also estimated the results including proxy respondents (Supplementary Appendix Table 10) and excluding imputed word recall scores (Supplementary Appendix Table 11).

Because the availability of body mass index in ELSA reduces the available common sample for models M1–M4 in a nonnegligible way, we compared results for models M1 and M2 based on a sample that does not have this data restriction (Supplementary Appendix Table 12).

To explore potential differences in different memory processes, we examined immediate and delayed recall separately (Supplementary Appendix Tables 13 and 14).

Our preferred results are based on random-slope mixed models that account for dependence of observations within individuals. We have compared the results to naive OLS and logistic models that assume independence and to models that estimate clustered standard errors (Supplementary Appendix Table 17 for clustered OLS; the naive OLS and logistic tables are available upon request). We have also compared results to a specification that only includes a random intercept but not a random age slope (Supplementary Appendix Table 18).

There may be substantially different attrition across the surveys, differential pursuit of self-interviews, and the use of proxy respondents to estimate memory impairment (29). In particular, attrition may be NMAR, that is, dropout depends on unobservables. One of the few methods that can deal with all of the above are joint models that model a longitudinal outcome (here: cognition) along with a survival process. Ideally, the survival processes that include both mortality and nonresponse/attrition would be modeled separately. Such models are possible in principle (30), but for our large data sets currently computationally unfeasible. In order to check for the influence of differential mortality, we run a standard joint model where the failure event is death. We use either model M1 or M2 as the longitudinal submodel. The survival submodel uses a Gompertz hazard and contains a similar covariate list as either model M1 or M2, the only exception being the indicator variable for first test occasion, which is not included. The 2 submodels are linked via the impact of the 2 common random effects (intercept and slope) on the survival submodel. The covariance between the 2 random effects is again unrestricted. Supplementary Appendix Table 19 shows that the results so obtained are very similar to our main results, which do not specify a mortality process. In further analyses, in order to rule out biased estimates through differential attrition, we redefine our failure event to comprise both death and attrition. In a first definition, failure is either end-of-trajectory nonresponse for 2 contiguous waves for subjects whose death is not known, or death. This yields very similar parameter estimates to the previous failure definition (Supplementary Appendix Table 20). A further modification that gives precedence to a presumed dropout (nonresponse) over (later) death of a given trajectory yields estimates so similar that it did not merit its own appendix table (results are available upon request).

## Discussion

All countries that were analyzed experienced improving trends in memory function. The pace of progress, however, varies widely, and the trend for the United States stands out in the international comparison in that it is much less steep than that of other countries. Although others have also documented improvements in various measures of population-level cognitive function in the United States (31,32), the current study indicates that this improvement is much shallower than in peer nations.

Our finding on the trends in the United States being less positive than in other countries is not unique to memory function. Prior research has documented concerning trends for subsets of the United States population for physical health outcomes. In a landmark paper on mortality, Case and Deaton (33) showed that, whereas most peer countries have experienced declines in mortality, for the United States, mortality for White men aged 45–54 increased over the years 1999–2013. Since then, several alarming reports have contributed to the picture of American exceptionalism. The adverse trends in mortality are not isolated to one subpopulation, but are affecting the U.S. population more broadly, and since 2010, they have led to stagnating and in some years even decreasing life expectancy (34), even before the impact of COVID-19. This end-of-progress against mortality is attributable to a multitude of forces, including rapidly increasing mortality from drug-related causes (35) and stagnation of progress in cardiovascular disease mortality (36).

Cardiometabolic conditions—obesity, diabetes, hypertension, heart disease, heart attacks, and stroke—may be key in understanding the U.S. divergence from improving memory trends, as they amplify the risk of ADRD (22). The United States has higher prevalence of obesity and cardiometabolic health conditions than the comparison countries, which may explain some of the adverse trends and offset the positive force of increasing educational distribution. We controlled for a large set of these risk factors, but that did not meaningfully change the country rankings. However, the measures that were available in this study were perhaps too crude to fully account for the adverse cardiometabolic health profile that differentiates the United States from peer countries. For example, prior research on obesity as a risk factor for physical health outcomes suggests that exposure to obesity over the life course would be preferred over BMI at survey (37), the measure that was available for this study.

Despite the U.S. poor performance in comparative perspective, the ubiquitously improving trend, even the weakly positive trend in the United States, is positive news. That said, the improvement in the United States is mostly driven by the last observation points, and based on this study, it was not possible to evaluate whether these last data points are outliers or whether they signal a change in trend from little improvement to rapid improvement. Our last observation for the United States was the year 2018, and subsequent data points starting in 2020 would cover the COVID-19 years during which both memory function and measurements may have changed in a special way, complicating the analysis.

Overall, across countries, we observe that education is among the primary factors for positive trends in memory function (Models 2 and 4, Tables 2 and 3). This is expected, as a substantial body of research documents the positive association between education and cognitive reserve (38). However, education is not the sole explanation. Between-country differences in both trends and social and contextual factors could be further exploited to help identify other mechanisms that may vary, such as increased access to nutrition and healthcare.

Analyses by population subgroup revealed important differences. The regional differences—robust improvement in England and SHARE countries, weak improvement in the United States—were largely the same when data were analyzed separately for men and women or by educational status. An important deviation from this overall pattern were the results by age. The gap between the United States and other countries is largest at ages 50–64; the gap narrows in age group 65–74; and in the age group 75 and older, the United States has a positive time trend that is no longer the weakest but matches that of England. This is clearly positive news, as the broader individual and societal implications of hampered memory function are more relevant at older than younger ages. A potential emerging concern is whether the current younger cohorts in the United States—whose trend in memory function is particularly weak compared to other countries—will carry with them into the future this weak trend.

These remaining questions point to some of the limitations of the study. Although considerable effort has been invested in harmonizing these 3 sister studies (15) and measuring cognitive function consistently across countries and across time, it is likely there remain some inconsistencies. Because we are analyzing within-country time trends, the between-country variation is less problematic than measurement changes over time. For example, the HRS increased efforts across waves to have respondents complete the TICS: The share of proxy interviews, hovering at around 10%–11% for years 1996–2002, declined thereafter from about 9% (2004) to about 4.5% (2018). This could bias the U.S. memory function trend downward compared to the other surveys (29). Although we ran additional robustness tests to analyze the potential role of panel conditioning and attrition, this effect cannot be excluded. However, when we ran comparable binary outcome regressions, one with and one without proxy respondents, we found no such effect: The trend coefficient worsened instead of improving (Supplementary Appendix Table 10, lines 3, 9, and 10). Note that the inclusion of proxy respondents is easily achieved (and customary) in binary outcome regressions for the full TICS interview of the HRS since there exists a validated mapping of proxy scores to self-respondent TICS scores. However, there is no such straightforward procedure for continuous cognitive scores.

Although SHARE-11 has a fairly flat time profile of missing memory scores due to proxy interviews of 2%–3%, in ELSA the share of proxy respondents has also changed over time. It has increased from about 2% in early waves to about 6% in later waves. Differently than the HRS, ELSA unfortunately does not have an established procedure for assessing cognitive function using both self-respondent and proxy interviews. We therefore refrain from conducting an analysis for ELSA similar to the one for the HRS that was described in the previous paragraph.

The 3 surveys also have differential coverage of residential care and nursing home residents, with HRS having the best coverage, SHARE coming second, and ELSA having the lowest coverage. Higher coverage of residential care and nursing home residents could possibly explain part of the differences in the trends between HRS and other surveys. We also estimated the results after excluding all residential care and nursing home residents (Supplementary Appendix Table 15). The results changed only little, suggesting that differential coverage of residential care and nursing home residents is not driving the country differences. However, it is likely there are differences for which we cannot account. Further, more refined measures on cardiometabolic risk factors, preferably over the life course, would be useful when exploring the mechanisms behind the country-to-country variation; unfortunately, these are not available across the data sets.

Our study focuses on memory function, measured by word recall, and interpretations regarding cognitive function more generally must be cautious. Much of the literature on trends in cognitive impairment that is based on the HRS uses 4 indices from the TICS-M that have been consistently measured across the widest time span and for all participants (ie, not restricted to those age 65+): immediate word recall, delayed word recall, backwards counting, and serial 7s (31,39). In the case of comparing the HRS, ELSA, and SHARE, all 4 indices were not available consistently. However, word recall comprises 20 out of 27 of the points on that standard 0–27 scale, and sensitivity analyses for HRS data in which we compare predictions of dementia using the 2 components versus the 4 components (see Supplementary Appendix Table 10) show very little difference. In sum, although analyzing long-term trends across these large, longitudinal data sets prevents us from using broad measures of cognitive function, these indicators of episodic memory are likely indicative of cognitive function.

A further limitation is that all regressions are unweighted. Survey weights can account for sampling probabilities (including oversampling) and selective nonresponse and attrition. Unfortunately, both SHARE and ELSA only provide longitudinal weights for a fully balanced panel, that is, a subset of respondents that responded in every survey wave. This makes these weights unsuitable for our trend analysis, which must include new subject additions to and subject removals from the sample. We note that the covariates in our regressions at least partially account for any relevant oversampling in the 3 surveys (eg, inclusion of a covariate for race/ethnicity for the HRS). In order to also address selective attrition, we used joint models as a robustness check.

Strengths of this paper include the use of 3 high-quality, population-based panel surveys, all of which use consistent measurement of memory function—immediate and delayed recall—that is understood to identify neurophysiological decline, as well as harmonized education, demographic, behavioral, and health measures (15). We start by using mixed-effects linear regression models to analyze the average memory function score, exploring time as both categorical and continuous. To also investigate whether trends differ in the tail of the distribution, we then use mixed effects logistic regression models with a categorized measure of memory function and explore 4 different thresholds for impairment. We conduct several robustness checks to ensure that trends are not confounded by panel conditioning, attrition, or proxy responses.

This study contributes a novel comparative analysis of trends in memory function across the United States, England, and 11 European countries in SHARE. There are several implications from our findings. First, like others (3,4), we find primarily improving trends showing declining memory impairment and that trends in increasing educational attainment act as a possible mechanism. Second, the United States lagging behind from those trends in England and SHARE-11 is notable. The main goal of our analysis was to document the cross-country variation in trends in memory function, and as secondary goal explore the potential reasons. Despite a large number of potential mechanisms, we were not able to explain the cross-country variation in the time trends, and in particular, the mechanisms tested in this paper did not help us to understand the forces that are responsible for the comparatively weak performance of the United States. The most recent data points right before the breakout of COVID-19, however, suggest rapid improvement in memory function in the United States. Analyzing whether this positive trend has continued, and uncovering the causes of the cross-country heterogeneity should be both a research and public health priority.

## Supplementary Material

Supplementary data are available at *The Journals of Gerontology, Series A: Biological Sciences and Medical Sciences* online.

glae154_suppl_Supplementary_Appendix
