# Supplementary material for: Trends in Memory Function and Memory Impairment Among Older Adults in the United States and Europe, 1996–2018
Source: J Gerontol A Biol Sci Med Sci. 2024 Nov 7;79(Suppl 1):S11–21. doi: 10.1093/gerona/glae154 (PMC11542220; doi:10.1093/gerona/glae154)
Supplement: glae154_suppl_Supplementary_Appendix [file glae154_suppl_supplementary_appendix.docx]

Supplementary **Supplemental Material (Appendix)**

List of Appendix Tables and Appendix Figures

[Appendix Tables 3](#_Toc168743306)

[Appendix Table 1A. Descriptive statistics by wave-pair for all waves: HRS 3](#_Toc168743307)

[Appendix Table 1B. Descriptive statistics by wave-pair for all waves: ELSA 4](#_Toc168743308)

[Appendix Table 1C. Descriptive statistics by wave-pair for all waves: SHARE-11 5](#_Toc168743309)

[Appendix Table 2A. Full regression results of models M1-M4 for the HRS 6](#_Toc168743310)

[Appendix Table 2B. Full regression results of models M1-M4 for ELSA 7](#_Toc168743311)

[Appendix Table 2C. Full regression results of models M1-M4 for SHARE-11 8](#_Toc168743312)

[Appendix Table 3A. Change in continuous memory score over 10 years, by subsamples of gender, education, and age group: HRS and ELSA 9](#_Toc168743313)

[Appendix Table 3B. Change in continuous memory score over 10 years, by subsamples of gender, education, and age group: , Aggregate SHARE-11 and Individual SHARE countries. 10](#_Toc168743314)

[Appendix Table 4. Odds ratios from logistic regressions for 10-year change in memory impairment, models M1-M4 11](#_Toc168743315)

[Appendix Table 5. Linear time trend coefficients for continuous score, models M1-M4: No adjustment for practice effects 12](#_Toc168743316)

[Appendix Table 6. Odds ratios from logistic regressions for 10-year change in memory impairment, models M1-M4: No adjustment for practice effects 13](#_Toc168743317)

[Appendix Table 7. Linear time trend coefficients for continuous score, models M1-M4: Age adjustment by 5-year age groups 14](#_Toc168743318)

[Appendix Table 8. Linear time trend coefficients for continuous score, models M1-M4: Practice effect measure is categorical variable. 15](#_Toc168743319)

[Appendix Table 9. Linear time trend coefficients for continuous score, models M1-M4: Samples restricted to first-time test takers. 16](#_Toc168743320)

[Appendix Table 10. Comparison of main logistic HRS regressions to Hale et al. (2020) 17](#_Toc168743321)

[Appendix Table 11. Main linear HRS regressions exclusive of observations with imputed word recall scores 18](#_Toc168743322)

[Appendix Table 12. Comparison of ELSA regressions when sample is not restricted by the availability of BMI data 19](#_Toc168743323)

[Appendix Table 13. Linear time trend coefficients for continuous score, models M1-M4: Dependent variable is immediate recall score 20](#_Toc168743324)

[Appendix Table 14. Linear time trend coefficients for continuous score, models M1-M4: Dependent variable is delayed recall score 21](#_Toc168743325)

[Appendix Table 15. Linear time trend coefficients for continuous score, models M1-M4: Sample excluding nursing home residents 22](#_Toc168743326)

[Appendix Table 16. Linear time trend coefficients for continuous score, models M1-M4: Sample restricted to ages 60 and over 23](#_Toc168743327)

[Appendix Table 17. Linear time trend coefficients for continuous score, models M1-M4: Least squares regression 24](#_Toc168743328)

[Appendix Table 18. Linear time trend coefficients for continuous score, models M1-M4: No random age slopes 25](#_Toc168743329)

[Appendix Table 19. Linear time trend coefficients for continuous score, models M1 and M2: Joint models for memory outcome and mortality 26](#_Toc168743330)

[Appendix Table 20. Linear time trend coefficients for continuous score, models M1and M2: Joint models for memory outcome and attrition/mortality 27](#_Toc168743331)

[Appendix Figures 28](#_Toc168743332)

[Appendix Figure 1. Time trend with confidence bounds for continuous score models M1 28](#_Toc168743333)

[Appendix Figure 2. Change in binary memory impairment over 10 years, by subsamples of gender, education, and age group 30](#_Toc168743334)

[Appendix Figure 3. Odds ratios for memory impairment of wave year indicator variables (model M1) 31](#_Toc168743335)

[References 32](#_Toc168743336)

# Appendix Tables

Appendix Table 1A. Descriptive statistics by wave-pair for all waves: HRS

|  | **1996-1998** | **2000-2002** | **2004-2006** | **2008-2010** | **2012-2014** | **2016-2018** | **All years** |
| --- | --- | --- | --- | --- | --- | --- | --- |
| Age (years, mean) | 66 | 68 | 68 | 67 | 68 | 67 | 67 |
| Women (%) | 59 | 60 | 59 | 59 | 59 | 58 | 59 |
| Memory function score (mean; range 0-20) | 10.0 | 9.9 | 9.7 | 9.6 | 9.6 | 9.8 | 9.8 |
| Memory impairment (1.5 SD) | 13.2 | 12.3 | 12.5 | 12.4 | 12.4 | 11.1 | 12.3 |
| Number of prior tests (mean) | 1.4 | 3.1 | 4.4 | 5.3 | 6.2 | 6.4 | 4.5 |
| Educational attainment |  |  |  |  |  |  |  |
| Less than upper secondary | 28 | 24 | 21 | 19 | 18 | 17 | 21 |
| Upper secondary / vocational training | 55 | 57 | 58 | 59 | 59 | 59 | 58 |
| Tertiary education | 17 | 19 | 21 | 22 | 23 | 24 | 21 |
| Obesity |  |  |  |  |  |  |  |
| Underweight (BMI <18.5) | 2 | 1 | 1 | 1 | 1 | 1 | 1 |
| Normal (BMI >=18.5, <25) | 37 | 35 | 34 | 31 | 29 | 27 | 32 |
| Overweight (BMI >=25, <30) | 40 | 41 | 40 | 40 | 39 | 38 | 40 |
| Obese (BMI >=30) | 21 | 23 | 25 | 28 | 31 | 34 | 27 |
| Smoking |  |  |  |  |  |  |  |
| Never | 41 | 41 | 43 | 43 | 44 | 46 | 43 |
| Former | 42 | 44 | 43 | 43 | 42 | 40 | 42 |
| Current | 17 | 14 | 14 | 14 | 14 | 14 | 15 |
| Ever diagnosed health conditions |  |  |  |  |  |  |  |
| Diabetes | 13 | 15 | 18 | 21 | 24 | 28 | 20 |
| High blood pressure | 43 | 48 | 53 | 57 | 61 | 62 | 54 |
| Heart problems | 20 | 22 | 23 | 23 | 24 | 24 | 23 |
| Stroke | 6 | 7 | 8 | 8 | 9 | 9 | 8 |
| Arthritis | 48 | 54 | 56 | 57 | 58 | 58 | 55 |
| Cancer | 10 | 12 | 13 | 14 | 15 | 15 | 13 |
| Lung disease | 7 | 8 | 8 | 9 | 10 | 11 | 9 |
| Psychological problems | 9 | 11 | 14 | 16 | 19 | 21 | 15 |
| Partnered | 67 | 65 | 64 | 63 | 62 | 59 | 63 |
| Exercises >=1/week | 44 | 42 | 54 | 50 | 48 | 49 | 48 |
| Born in foreign country | 9 | 9 | 10 | 12 | 14 | 16 | 12 |
| Person-waves | 33,470 | 32,382 | 33,894 | 35,005 | 35,437 | 33,212 | 203,400 |

Notes: Descriptive statistics for all wave-pairs of the HRS. Notes from Table 1 apply.

Appendix Table 1B. Descriptive statistics by wave-pair for all waves: ELSA

|  | **1996-1998** | **2000-2002** | **2004-2006** | **2008-2010** | **2012-2014** | **2016-2018** | **All years** |
| --- | --- | --- | --- | --- | --- | --- | --- |
| Age (years, mean) | - | 64 | 66 | 66 | 68 | 71 | 67 |
| Women (%) | - | 53 | 54 | 54 | 54 | 55 | 54 |
| Memory function score (mean; range 0-20) | - | 9.9 | 10.2 | 10.5 | 10.6 | 10.6 | 10.4 |
| Memory impairment (1.5 SD) | - | 16.4 | 15.0 | 13.7 | 13.2 | 14.3 | 14.3 |
| Number of prior tests (mean) | - | 1.0 | 2.4 | 3.5 | 5.1 | 7.0 | 4.0 |
| Educational attainment |  |  |  |  |  |  |  |
| Less than upper secondary | - | 41 | 38 | 33 | 29 | 26 | 33 |
| Upper secondary / vocational training | - | 45 | 47 | 49 | 51 | 53 | 49 |
| Tertiary education | - | 14 | 15 | 18 | 20 | 21 | 18 |
| Obesity |  |  |  |  |  |  |  |
| Underweight (BMI <18.5) | - | 1 | 1 | 1 | 1 | 1 | 1 |
| Normal (BMI >=18.5, <25) | - | 27 | 27 | 26 | 27 | 27 | 27 |
| Overweight (BMI >=25, <30) | - | 43 | 43 | 42 | 42 | 43 | 42 |
| Obese (BMI >=30) | . | 30 | 30 | 31 | 31 | 30 | 30 |
| Smoking | - |  |  |  |  |  |  |
| Never | - | 36 | 37 | 39 | 37 | 37 | 37 |
| Former | - | 47 | 48 | 48 | 52 | 54 | 50 |
| Current | . | 17 | 15 | 13 | 11 | 9 | 13 |
| Ever diagnosed health conditions | - |  |  |  |  |  |  |
| Diabetes | - | 6 | 8 | 10 | 11 | 13 | 10 |
| High blood pressure | - | 35 | 41 | 41 | 43 | 47 | 42 |
| Heart problems | - | 14 | 17 | 17 | 20 | 26 | 19 |
| Stroke | - | 3 | 4 | 4 | 5 | 6 | 4 |
| Arthritis | - | 29 | 34 | 36 | 39 | 44 | 37 |
| Cancer | - | 5 | 7 | 9 | 12 | 15 | 10 |
| Lung disease | - | 5 | 6 | 6 | 7 | 8 | 6 |
| Psychological problems | - | 7 | 8 | 9 | 11 | 12 | 10 |
| Partnered | - | 72 | 70 | 71 | 70 | 68 | 70 |
| Exercises >=1/week | - | 61 | 62 | 61 | 62 | 62 | 62 |
| Born in foreign country | - | 6 | 6 | 8 | 9 | 9 | 8 |
| Person-waves | - | 7,239 | 14,309 | 16,296 | 15,102 | 11,712 | 64,659 |

Notes: Descriptive statistics for all wave-pairs of ELSA. Notes from Table 1 apply.

Appendix Table 1C. Descriptive statistics by wave-pair for all waves: SHARE-11

|  | **1996-1998** | **2000-2002** | **2004-2006** | **2008-2010** | **2012-2014** | **2016-2018** | **All years** |
| --- | --- | --- | --- | --- | --- | --- | --- |
| Age (years, mean) | - | - | 65 | 66 | 67 | 70 | 67 |
| Women (%) | - | - | 55 | 55 | 55 | 56 | 55 |
| Memory function score (mean; range 0-20) | - | - | 8.4 | 9.2 | 9.3 | 9.2 | 9.1 |
| Memory impairment (1.5 SD) | - | - | 16.8 | 14.8 | 13.5 | 13.5 | 14.4 |
| Number of prior tests (mean) | - | - | 1.4 | 1.8 | 2.5 | 4.2 | 2.6 |
| Educational attainment |  |  |  |  |  |  |  |
| Less than upper secondary | - | - | 49 | 42 | 40 | 38 | 42 |
| Upper secondary / vocational training | - | - | 31 | 36 | 35 | 36 | 35 |
| Tertiary education | - | - | 20 | 22 | 25 | 26 | 24 |
| Obesity |  |  |  |  |  |  |  |
| Underweight (BMI <18.5) | - | - | 1 | 1 | 1 | 1 | 1 |
| Normal (BMI >=18.5, <25) | - | - | 38 | 39 | 39 | 39 | 39 |
| Overweight (BMI >=25, <30) | - | - | 43 | 41 | 41 | 42 | 42 |
| Obese (BMI >=30) | . | . | 18 | 18 | 19 | 19 | 18 |
| Smoking | - | - |  |  |  |  |  |
| Never | - | - | 54 | 52 | 53 | 55 | 54 |
| Former | - | - | 28 | 30 | 29 | 30 | 29 |
| Current | . | . | 18 | 18 | 17 | 15 | 17 |
| Ever diagnosed health conditions | - | - |  |  |  |  |  |
| Diabetes | - | - | 11 | 12 | 14 | 17 | 14 |
| High blood pressure | - | - | 37 | 40 | 46 | 55 | 45 |
| Heart problems | - | - | 14 | 14 | 16 | 20 | 16 |
| Stroke | - | - | 4 | 5 | 6 | 8 | 6 |
| Arthritis | - | - | 24 | 30 | 35 | 45 | 35 |
| Cancer | - | - | 6 | 8 | 10 | 13 | 9 |
| Lung disease | - | - | 6 | 8 | 9 | 11 | 9 |
| Psychological problems | - | - | 9 | 11 | 13 | 17 | 13 |
| Partnered | - | - | 75 | 75 | 75 | 73 | 75 |
| Exercises >=1/week | - | - | 70 | 69 | 69 | 68 | 69 |
| Born in foreign country | - | - | 11 | 9 | 10 | 9 | 10 |
| Person-waves | - | - | 43,871 | 30,552 | 82,874 | 55,477 | 212,776 |

Notes: Descriptive statistics for all wave-pairs of SHARE. Notes from Table 1 apply.

Appendix Table 2A. Full regression results of models M1-M4 for the HRS

|  | **M1 - descriptive** | | **M2 - education** | | **M3 - health, demographic** | | **M4 - full adjustment** | |
| --- | --- | --- | --- | --- | --- | --- | --- | --- |
|  | **b** | **95% CI** | **b** | **95% CI** | **b** | **95% CI** | **b** | **95% CI** |
| Linear time trend (unit: 10 years) | 0.06 | [0.04,0.09] | -0.13 | [-0.15,-0.10] | 0.15 | [0.13,0.18] | -0.05 | [-0.07,-0.02] |
| Demographic |  |  |  |  |  |  |  |  |
| Male | -0.87 | [-0.93,-0.82] | -0.93 | [-0.99,-0.88] | -0.91 | [-0.97,-0.85] | -0.99 | [-1.04,-0.93] |
| Partnered |  |  |  |  | 0.17 | [0.13,0.20] | 0.13 | [0.09,0.16] |
| Foreign born |  |  |  |  | -0.24 | [-0.33,-0.15] | -0.13 | [-0.22,-0.05] |
| Age (linear term; unit: 10 years; centered at 50) | 0.03 | [-0.02,0.09] | 0.20 | [0.15,0.25] | 0.06 | [0.01,0.12] | 0.22 | [0.16,0.27] |
| Age (quadratic term; unit: 10 years; centered at 50) | -0.40 | [-0.42,-0.39] | -0.40 | [-0.41,-0.39] | -0.38 | [-0.40,-0.37] | -0.38 | [-0.40,-0.37] |
| Education (ref: < upper sec.) |  |  |  |  |  |  |  |  |
| Upp sec./voc. train. |  |  | 1.66 | [1.60,1.73] |  |  | 1.55 | [1.49,1.62] |
| Tertiary education |  |  | 2.99 | [2.92,3.07] |  |  | 2.78 | [2.71,2.86] |
| Test # |  |  |  |  |  |  |  |  |
| 1 | -0.10 | [-0.13,-0.06] | -0.10 | [-0.13,-0.06] | -0.12 | [-0.15,-0.08] | -0.11 | [-0.15,-0.08] |
| Obesity (ref.: Normal) |  |  |  |  |  |  |  |  |
| Underweight |  |  |  |  | -0.44 | [-0.68,-0.21] | -0.32 | [-0.54,-0.10] |
| Overweight |  |  |  |  | -0.01 | [-0.07,0.05] | 0.07 | [0.02,0.13] |
| Obese |  |  |  |  | -0.08 | [-0.15,-0.01] | 0.06 | [-0.00,0.13] |
| Behaviors |  |  |  |  |  |  |  |  |
| Former |  |  |  |  | -0.19 | [-0.25,-0.14] | -0.06 | [-0.11,-0.00] |
| Current |  |  |  |  | -0.42 | [-0.48,-0.35] | -0.14 | [-0.20,-0.07] |
| Exercises >1/week |  |  |  |  | 0.27 | [0.24,0.29] | 0.24 | [0.21,0.27] |
| Health: ever diagnosed with: |  |  |  |  |  |  |  |  |
| Diabetes |  |  |  |  | -0.21 | [-0.26,-0.16] | -0.17 | [-0.22,-0.12] |
| High blood pressure |  |  |  |  | -0.10 | [-0.14,-0.06] | -0.07 | [-0.11,-0.04] |
| Heart problems |  |  |  |  | -0.14 | [-0.19,-0.10] | -0.11 | [-0.15,-0.07] |
| Stroke |  |  |  |  | -0.74 | [-0.81,-0.67] | -0.70 | [-0.76,-0.63] |
| Arthritis |  |  |  |  | -0.08 | [-0.12,-0.04] | -0.02 | [-0.06,0.02] |
| Cancer |  |  |  |  | 0.04 | [-0.01,0.10] | 0.01 | [-0.04,0.07] |
| Lung disease |  |  |  |  | -0.20 | [-0.26,-0.13] | -0.09 | [-0.15,-0.03] |
| Psychological problems |  |  |  |  | -0.54 | [-0.60,-0.49] | -0.50 | [-0.55,-0.44] |
| Model statistic |  |  |  |  |  |  |  |  |
| Number of observations | 203,400 |  | 203,400 |  | 203,400 |  | 203,400 |  |

Notes: Full results for models M1-M4 using HRS data. Standardized time coefficients of these models have been shown in Table 2. The numbers are based on linear regressions on quasi-continuous memory score 0-20. M1 regresses memory score on calendar year and includes controls for age, gender, number of tests taken, and (not shown) race/ethnicity and interview mode. M2 adds education. M3 adds demographic variables (migration, partnership), behaviors, and underlying health conditions to M1. M4 adds education to model M3. Time coefficients in this table are not standardized.

Appendix Table 2B. Full regression results of models M1-M4 for ELSA

|  | **M1 - descriptive** | | **M2 - education** | | **M3 - health, demographic** | | **M4 - full adjustment** | |
| --- | --- | --- | --- | --- | --- | --- | --- | --- |
|  | **b** | **95% CI** | **b** | **95% CI** | **b** | **95% CI** | **b** | **95% CI** |
| Linear time trend (unit: 10 years) | 0.67 | [0.60,0.73] | 0.34 | [0.27,0.40] | 0.73 | [0.66,0.79] | 0.41 | [0.34,0.47] |
| Demographic |  |  |  |  |  |  |  |  |
| Male | -0.72 | [-0.82,-0.62] | -1.00 | [-1.09,-0.91] | -0.73 | [-0.82,-0.63] | -1.00 | [-1.10,-0.91] |
| Partnered |  |  |  |  | 0.07 | [-0.01,0.15] | 0.02 | [-0.06,0.10] |
| Foreign born |  |  |  |  | -0.68 | [-0.86,-0.51] | -0.84 | [-1.01,-0.68] |
| Age (linear term; unit: 10 years; centered at 50) | 0.11 | [0.01,0.22] | 0.43 | [0.33,0.54] | 0.06 | [-0.05,0.16] | 0.36 | [0.26,0.47] |
| Age (quadratic term; unit: 10 years; centered at 50) | -0.49 | [-0.52,-0.47] | -0.49 | [-0.52,-0.47] | -0.46 | [-0.49,-0.43] | -0.46 | [-0.49,-0.44] |
| Education (ref: < upper sec.) |  |  |  |  |  |  |  |  |
| Upp sec./voc. train. |  |  | 1.73 | [1.63,1.84] |  |  | 1.64 | [1.53,1.74] |
| Tertiary education |  |  | 2.97 | [2.83,3.10] |  |  | 2.85 | [2.72,2.99] |
| Test # |  |  |  |  |  |  |  |  |
| 1 | -0.35 | [-0.41,-0.29] | -0.35 | [-0.41,-0.29] | -0.35 | [-0.41,-0.29] | -0.35 | [-0.41,-0.29] |
| Obesity (ref.: Normal) |  |  |  |  |  |  |  |  |
| Underweight |  |  |  |  | -0.91 | [-1.45,-0.37] | -0.94 | [-1.44,-0.44] |
| Overweight |  |  |  |  | -0.11 | [-0.23,0.01] | 0.02 | [-0.09,0.13] |
| Obese |  |  |  |  | -0.47 | [-0.59,-0.34] | -0.17 | [-0.29,-0.05] |
| Behaviors |  |  |  |  |  |  |  |  |
| Former |  |  |  |  | -0.17 | [-0.26,-0.08] | -0.06 | [-0.15,0.02] |
| Current |  |  |  |  | -0.47 | [-0.59,-0.35] | -0.17 | [-0.29,-0.06] |
| Exercises >1/week |  |  |  |  | 0.30 | [0.25,0.35] | 0.25 | [0.21,0.30] |
| Health: ever diagnosed with: |  |  |  |  |  |  |  |  |
| Diabetes |  |  |  |  | -0.28 | [-0.40,-0.17] | -0.25 | [-0.36,-0.14] |
| High blood pressure |  |  |  |  | -0.07 | [-0.14,0.00] | -0.04 | [-0.11,0.03] |
| Heart problems |  |  |  |  | -0.06 | [-0.15,0.03] | -0.06 | [-0.14,0.03] |
| Stroke |  |  |  |  | -0.88 | [-1.04,-0.72] | -0.82 | [-0.97,-0.66] |
| Arthritis |  |  |  |  | -0.04 | [-0.12,0.03] | 0.01 | [-0.06,0.08] |
| Cancer |  |  |  |  | 0.01 | [-0.10,0.12] | -0.03 | [-0.14,0.07] |
| Lung disease |  |  |  |  | -0.23 | [-0.37,-0.09] | -0.09 | [-0.23,0.04] |
| Psychological problems |  |  |  |  | -0.25 | [-0.38,-0.12] | -0.29 | [-0.41,-0.16] |
| Model statistic |  |  |  |  |  |  |  |  |
| Number of observations | 64,658 |  | 64,658 |  | 64,658 |  | 64,658 |  |

Notes: Full results for models M1-M4 using ELSA data. Notes from Appendix Table 2A apply, with the exception that M1 does not adjust for race/ethnicity and interview mode.

Appendix Table 2C. Full regression results of models M1-M4 for SHARE-11

|  | **M1 - descriptive** | | **M2 - education** | | **M3 - health, demographic** | | **M4 - full adjustment** | |
| --- | --- | --- | --- | --- | --- | --- | --- | --- |
|  | **b** | **95% CI** | **b** | **95% CI** | **b** | **95% CI** | **b** | **95% CI** |
| Linear time trend (unit: 10 years) | 0.89 | [0.86,0.93] | 0.63 | [0.59,0.67] | 0.99 | [0.95,1.02] | 0.72 | [0.68,0.75] |
| Demographic |  |  |  |  |  |  |  |  |
| Male | -0.52 | [-0.56,-0.48] | -0.70 | [-0.74,-0.66] | -0.59 | [-0.64,-0.55] | -0.78 | [-0.82,-0.74] |
| Partnered |  |  |  |  | 0.15 | [0.11,0.19] | 0.16 | [0.11,0.20] |
| Foreign born |  |  |  |  | -0.12 | [-0.19,-0.05] | -0.40 | [-0.47,-0.34] |
| Age (linear term; unit: 10 years; centered at 50) | -0.24 | [-0.29,-0.18] | -0.01 | [-0.07,0.04] | -0.26 | [-0.31,-0.20] | -0.05 | [-0.11,0.00] |
| Age (quadratic term; unit: 10 years; centered at 50) | -0.38 | [-0.39,-0.36] | -0.37 | [-0.38,-0.35] | -0.33 | [-0.35,-0.32] | -0.32 | [-0.34,-0.31] |
| Education (ref: < upper sec.) |  |  |  |  |  |  |  |  |
| Upp sec./voc. train. |  |  | 1.73 | [1.68,1.77] |  |  | 1.64 | [1.59,1.68] |
| Tertiary education |  |  | 2.76 | [2.71,2.81] |  |  | 2.64 | [2.58,2.69] |
| Test # |  |  |  |  |  |  |  |  |
| 1 | -0.34 | [-0.36,-0.31] | -0.33 | [-0.36,-0.30] | -0.38 | [-0.40,-0.35] | -0.36 | [-0.39,-0.33] |
| Obesity (ref.: Normal) |  |  |  |  |  |  |  |  |
| Underweight |  |  |  |  | -0.17 | [-0.37,0.02] | -0.21 | [-0.40,-0.03] |
| Overweight |  |  |  |  | -0.34 | [-0.38,-0.29] | -0.15 | [-0.19,-0.11] |
| Obese |  |  |  |  | -0.53 | [-0.59,-0.47] | -0.22 | [-0.28,-0.17] |
| Behaviors |  |  |  |  |  |  |  |  |
| Former |  |  |  |  | 0.36 | [0.32,0.41] | 0.28 | [0.24,0.32] |
| Current |  |  |  |  | 0.13 | [0.08,0.18] | 0.15 | [0.10,0.20] |
| Exercises >1/week |  |  |  |  | 0.47 | [0.44,0.50] | 0.42 | [0.39,0.45] |
| Health: ever diagnosed with: |  |  |  |  |  |  |  |  |
| Diabetes |  |  |  |  | -0.45 | [-0.50,-0.40] | -0.35 | [-0.40,-0.31] |
| High blood pressure |  |  |  |  | -0.11 | [-0.14,-0.07] | -0.08 | [-0.11,-0.04] |
| Heart problems |  |  |  |  | -0.17 | [-0.22,-0.12] | -0.14 | [-0.19,-0.10] |
| Stroke |  |  |  |  | -0.63 | [-0.70,-0.56] | -0.66 | [-0.73,-0.59] |
| Arthritis |  |  |  |  | -0.13 | [-0.17,-0.10] | -0.07 | [-0.11,-0.04] |
| Cancer |  |  |  |  | 0.11 | [0.05,0.17] | 0.02 | [-0.04,0.08] |
| Lung disease |  |  |  |  | -0.24 | [-0.30,-0.18] | -0.17 | [-0.23,-0.12] |
| Psychological problems |  |  |  |  | -0.44 | [-0.49,-0.39] | -0.37 | [-0.42,-0.32] |
| Model statistic |  |  |  |  |  |  |  |  |
| Number of observations | 212,774 |  | 212,774 |  | 212,774 |  | 212,774 |  |

Notes: Full results for models M1-M4 using SHARE-11 data. Notes from Appendix Table 2A apply, with the exception that M1 does not adjust for race/ethnicity and interview mode.

Appendix Table 3A. Change in continuous memory score over 10 years, by subsamples of gender, education, and age group: HRS and ELSA

|  | **HRS** | | **ELSA** | | **HRS 2004-** | | **ELSA 2004-** | |
| --- | --- | --- | --- | --- | --- | --- | --- | --- |
|  | **b** | **95% CI** | **b** | **95% CI** | **b** | **95% CI** | b | 95% CI |
| Full sample | 0.02 | [0.01, 0.02] | 0.18 | [0.16, 0.20] | 0.04 | [0.03, 0.05] | 0.17 | [0.15, 0.19] |
| Gender |  |  |  |  |  |  |  |  |
| Men | 0.03 | [0.01, 0.04] | 0.18 | [0.15, 0.21] | 0.05 | [0.03, 0.06] | 0.17 | [0.14, 0.20] |
| Women | 0.01 | [0.00, 0.02] | 0.18 | [0.16, 0.21] | 0.03 | [0.02, 0.04] | 0.17 | [0.15, 0.20] |
| Age |  |  |  |  |  |  |  |  |
| 50-64 | -0.04 | [-0.05, -0.03] | 0.23 | [0.19, 0.26] | 0.00 | [-0.02, 0.02] | 0.20 | [0.16, 0.24] |
| 65-74 | 0.08 | [0.06, 0.10] | 0.31 | [0.27, 0.34] |  |  | 0.31 | [0.27, 0.35] |
| 75+ | 0.10 | [0.09, 0.12] | 0.10 | [0.06, 0.14] | 0.07 | [0.05, 0.09] | 0.10 | [0.06, 0.14] |
| Education |  |  |  |  |  |  |  |  |
| < Upp. secondary | -0.02 | [-0.04, -0.01] | 0.04 | [0.01, 0.07] | -0.01 | [-0.03, 0.02] | 0.03 | [0.00, 0.07] |
| Upp. sec./voc. train. | -0.05 | [-0.06, -0.04] | 0.11 | [0.09, 0.14] | -0.02 | [-0.03, -0.01] | 0.10 | [0.07, 0.13] |
| Tertiary | -0.01 | [-0.03, 0.00] | 0.15 | [0.11, 0.20] | 0.03 | [0.00, 0.05] | 0.15 | [0.10, 0.19] |

Notes: Standardized coefficient estimates as in Figure 2; see the figure note in the main text. Numbers in brackets are lower and upper bounds of 95% confidence intervals.

Appendix Table 3B. Change in continuous memory score over 10 years, by subsamples of gender, education, and age group: , Aggregate SHARE-11 and Individual SHARE countries.

|  | **SHARE** | | **Austria** | | **Belgium** | | **Denmark** | |
| --- | --- | --- | --- | --- | --- | --- | --- | --- |
|  | **b** | **95% CI** | **b** | **95% CI** | **b** | **95% CI** | **b** | **95% CI** |
| Full sample | 0.24 | [0.23, 0.25] | 0.17 | [0.13, 0.21] | 0.32 | [0.30, 0.35] | 0.09 | [0.05, 0.12] |
| Gender |  |  |  |  |  |  |  |  |
| Men | 0.25 | [0.23, 0.26] | 0.14 | [0.08, 0.21] | 0.35 | [0.31, 0.39] | 0.09 | [0.04, 0.15] |
| Women | 0.23 | [0.22, 0.25] | 0.19 | [0.14, 0.24] | 0.31 | [0.27, 0.34] | 0.08 | [0.04, 0.13] |
| Age |  |  |  |  |  |  |  |  |
| 50-64 | 0.29 | [0.27, 0.31] | 0.27 | [0.20, 0.35] | 0.39 | [0.34, 0.44] | 0.13 | [0.07, 0.18] |
| 65-74 | 0.30 | [0.28, 0.32] | 0.33 | [0.25, 0.41] | 0.38 | [0.32, 0.44] | 0.14 | [0.07, 0.22] |
| 75+ | 0.19 | [0.17, 0.22] | 0.13 | [0.05, 0.22] | 0.25 | [0.18, 0.32] | 0.04 | [-0.04, 0.13] |
| Education |  |  |  |  |  |  |  |  |
| < Upp. secondary | 0.19 | [0.17, 0.20] | 0.07 | [-0.01, 0.15] | 0.25 | [0.21, 0.30] | 0.06 | [-0.01, 0.14] |
| Upp. sec./voc. train. | 0.16 | [0.14, 0.18] | 0.13 | [0.07, 0.19] | 0.27 | [0.21, 0.32] | 0.03 | [-0.02, 0.09] |
| Tertiary | 0.20 | [0.18, 0.22] | 0.21 | [0.12, 0.30] | 0.32 | [0.27, 0.37] | 0.06 | [0.00, 0.12] |
|  | **France** | | **Germany** | | **Greece** | | **Israel** | |
|  | **b** | **95% CI** | **b** | **95% CI** | **b** | **95% CI** | **b** | **95% CI** |
| Full sample | 0.36 | [0.33, 0.39] | 0.21 | [0.17, 0.24] | 0.27 | [0.24, 0.30] | 0.42 | [0.37, 0.48] |
| Gender |  |  |  |  |  |  |  |  |
| Men | 0.38 | [0.33, 0.42] | 0.13 | [0.08, 0.19] | 0.29 | [0.25, 0.34] | 0.46 | [0.38, 0.54] |
| Women | 0.36 | [0.32, 0.39] | 0.27 | [0.22, 0.31] | 0.25 | [0.22, 0.29] | 0.40 | [0.33, 0.47] |
| Age |  |  |  |  |  |  |  |  |
| 50-64 | 0.50 | [0.44, 0.55] | 0.28 | [0.23, 0.34] | 0.33 | [0.28, 0.38] | 0.48 | [0.38, 0.58] |
| 65-74 | 0.40 | [0.33, 0.46] | 0.22 | [0.15, 0.28] | 0.31 | [0.24, 0.37] | 0.52 | [0.41, 0.62] |
| 75+ | 0.26 | [0.19, 0.33] | 0.14 | [0.06, 0.22] | 0.21 | [0.14, 0.28] | 0.36 | [0.23, 0.48] |
| Education |  |  |  |  |  |  |  |  |
| < Upp. secondary | 0.25 | [0.21, 0.30] | 0.03 | [-0.06, 0.13] | 0.33 | [0.29, 0.37] | 0.24 | [0.14, 0.33] |
| Upp. sec./voc. train. | 0.34 | [0.28, 0.40] | 0.19 | [0.15, 0.24] | 0.08 | [0.01, 0.14] | 0.41 | [0.31, 0.51] |
| Tertiary | 0.38 | [0.31, 0.45] | 0.23 | [0.17, 0.29] | 0.15 | [0.06, 0.23] | 0.43 | [0.33, 0.53] |
|  | **Italy** | | **Spain** | | **Sweden** | | **Switzerland** | |
|  | **b** | **95% CI** | **b** | **95% CI** | **b** | **95% CI** | **b** | **95% CI** |
| Full sample | 0.27 | [0.24, 0.30] | 0.36 | [0.33, 0.40] | 0.12 | [0.09, 0.15] | 0.30 | [0.26, 0.34] |
| Gender |  |  |  |  |  |  |  |  |
| Men | 0.32 | [0.27, 0.37] | 0.34 | [0.29, 0.39] | 0.10 | [0.05, 0.15] | 0.27 | [0.21, 0.34] |
| Women | 0.23 | [0.19, 0.27] | 0.38 | [0.34, 0.42] | 0.14 | [0.10, 0.19] | 0.33 | [0.27, 0.38] |
| Age |  |  |  |  |  |  |  |  |
| 50-64 | 0.28 | [0.22, 0.33] | 0.47 | [0.40, 0.53] | 0.13 | [0.07, 0.20] | 0.44 | [0.36, 0.53] |
| 65-74 | 0.31 | [0.25, 0.38] | 0.50 | [0.43, 0.57] | 0.15 | [0.09, 0.22] | 0.43 | [0.35, 0.51] |
| 75+ | 0.31 | [0.24, 0.38] | 0.25 | [0.18, 0.32] | 0.17 | [0.10, 0.24] | 0.21 | [0.11, 0.30] |
| Education |  |  |  |  |  |  |  |  |
| < Upp. secondary | 0.28 | [0.24, 0.32] | 0.33 | [0.30, 0.37] | 0.01 | [-0.04, 0.07] | 0.12 | [0.04, 0.20] |
| Upp. sec./voc. train. | 0.07 | [-0.00, 0.15] | 0.33 | [0.21, 0.46] | 0.04 | [-0.02, 0.11] | 0.27 | [0.22, 0.33] |
| Tertiary | 0.00 | [-0.13, 0.13] | 0.26 | [0.14, 0.38] | 0.13 | [0.06, 0.19] | 0.37 | [0.25, 0.49] |

Notes: See Appendix Figure 3A.

Appendix Table 4. Odds ratios from logistic regressions for 10-year change in memory impairment, models M1-M4

|  |  |  | **M1 - descriptive** | | **M2 - education** | | **M3 - health, demographic** | | **M4 - full adjustment** | |
| --- | --- | --- | --- | --- | --- | --- | --- | --- | --- | --- |
|  |  | **N** | **b** | **95% CI** | **b** | **95% CI** | **b** | **95% CI** | **b** | **95% CI** |
| **Survey** | HRS | 203,400 | 0.86 | [0.83, 0.89] | 1.03 | [0.99, 1.06] | 0.80 | [0.77, 0.83] | 0.96 | [0.92, 0.99] |
|  | ELSA | 64,658 | 0.67 | [0.61, 0.73] | 0.89 | [0.81, 0.97] | 0.64 | [0.58, 0.69] | 0.82 | [0.75, 0.90] |
|  | SHARE | 212,774 | 0.51 | [0.48, 0.53] | 0.59 | [0.56, 0.63] | 0.47 | [0.44, 0.49] | 0.55 | [0.52, 0.58] |
|  | HRS 2004- | 137,548 | 0.89 | [0.84, 0.94] | 1.02 | [0.96, 1.08] | 0.78 | [0.74, 0.83] | 0.91 | [0.86, 0.97] |
|  | ELSA 2004- | 57,419 | 0.68 | [0.61, 0.74] | 0.90 | [0.82, 0.99] | 0.64 | [0.58, 0.70] | 0.83 | [0.75, 0.91] |
|  | |  |  |  |  |  |  |  |  |  |
| **SHARE Countries** | Austria |  |  |  | 0.58 | [0.45, 0.70] | 0.48 | [0.37, 0.59] | 0.58 | [0.45, 0.71] |
|  | Belgium | 27,578 | 0.39 | [0.33, 0.45] | 0.50 | [0.42, 0.58] | 0.35 | [0.29, 0.40] | 0.45 | [0.38, 0.52] |
|  | Denmark | 18,316 | 0.87 | [0.69, 1.04] | 1.02 | [0.82, 1.23] | 0.86 | [0.68, 1.03] | 1.00 | [0.80, 1.20] |
|  | France | 22,964 | 0.37 | [0.30, 0.43] | 0.48 | [0.40, 0.56] | 0.36 | [0.30, 0.42] | 0.46 | [0.38, 0.54] |
|  | Germany | 21,454 | 0.69 | [0.56, 0.83] | 0.81 | [0.65, 0.96] | 0.64 | [0.51, 0.77] | 0.75 | [0.60, 0.89] |
|  | Greece | 14,313 | 0.59 | [0.50, 0.69] | 0.67 | [0.56, 0.78] | 0.49 | [0.41, 0.58] | 0.56 | [0.46, 0.65] |
|  | Israel | 9,531 | 0.39 | [0.28, 0.50] | 0.49 | [0.35, 0.63] | 0.32 | [0.23, 0.42] | 0.42 | [0.30, 0.54] |
|  | Italy | 22,945 | 0.46 | [0.38, 0.54] | 0.53 | [0.45, 0.62] | 0.41 | [0.35, 0.48] | 0.47 | [0.40, 0.55] |
|  | Spain | 23,306 | 0.41 | [0.35, 0.47] | 0.45 | [0.39, 0.52] | 0.38 | [0.33, 0.43] | 0.41 | [0.36, 0.47] |
|  | Sweden | 19,547 | 0.71 | [0.60, 0.81] | 0.87 | [0.74, 1.01] | 0.70 | [0.59, 0.81] | 0.85 | [0.71, 0.98] |
|  | Switzerland | 15,203 | 0.57 | [0.44, 0.69] | 0.75 | [0.58, 0.92] | 0.51 | [0.39, 0.62] | 0.67 | [0.52, 0.83] |

Notes: Based on survey/country-specific logistic regressions on binary impairment, defined by scores lower than 1.5 standard deviations below the mean for ages 50-69. Other than that, notes from Table 2 apply.

Appendix Table 5. Linear time trend coefficients for continuous score, models M1-M4:
No adjustment for practice effects

|  |  |  | **M1 - descriptive** | | **M2 - education** | | **M3 - health, demographic** | | **M4 - full adjustment** | |
| --- | --- | --- | --- | --- | --- | --- | --- | --- | --- | --- |
|  |  | **N** | **b** | **95% CI** | **b** | **95% CI** | **b** | **95% CI** | **b** | **95% CI** |
| **Survey** | HRS | 203,400 | 0.02 | [0.02, 0.03] | -0.03 | [-0.04, -0.02] | 0.05 | [0.04, 0.06] | -0.01 | [-0.01, 0.00] |
|  | ELSA | 64,658 | 0.22 | [0.21, 0.24] | 0.13 | [0.11, 0.15] | 0.24 | [0.22, 0.25] | 0.15 | [0.13, 0.17] |
|  | SHARE | 212,774 | 0.30 | [0.29, 0.31] | 0.22 | [0.22, 0.23] | 0.32 | [0.32, 0.33] | 0.25 | [0.24, 0.26] |
|  | HRS 2004- | 137,548 | 0.04 | [0.03, 0.05] | 0.00 | [-0.01, 0.01] | 0.08 | [0.07, 0.09] | 0.03 | [0.02, 0.04] |
|  | ELSA 2004- | 57,419 | 0.18 | [0.16, 0.19] | 0.08 | [0.07, 0.10] | 0.19 | [0.17, 0.21] | 0.10 | [0.09, 0.12] |
|  | |  |  |  |  |  |  |  |  |  |
| **SHARE Countries** | Austria | 17,617 | 0.31 | [0.27, 0.34] | 0.26 | [0.23, 0.30] | 0.31 | [0.27, 0.34] | 0.26 | [0.23, 0.30] |
|  | Belgium | 27,578 | 0.38 | [0.36, 0.41] | 0.32 | [0.30, 0.34] | 0.41 | [0.39, 0.44] | 0.35 | [0.32, 0.37] |
|  | Denmark | 18,316 | 0.16 | [0.13, 0.19] | 0.11 | [0.09, 0.14] | 0.18 | [0.15, 0.21] | 0.14 | [0.11, 0.17] |
|  | France | 22,964 | 0.41 | [0.39, 0.44] | 0.33 | [0.31, 0.36] | 0.43 | [0.40, 0.45] | 0.35 | [0.32, 0.37] |
|  | Germany | 21,454 | 0.29 | [0.26, 0.32] | 0.26 | [0.23, 0.28] | 0.32 | [0.29, 0.35] | 0.28 | [0.25, 0.31] |
|  | Greece | 14,313 | 0.24 | [0.22, 0.27] | 0.20 | [0.17, 0.22] | 0.27 | [0.24, 0.30] | 0.23 | [0.20, 0.25] |
|  | Israel | 9,531 | 0.45 | [0.41, 0.49] | 0.40 | [0.36, 0.44] | 0.48 | [0.44, 0.52] | 0.42 | [0.37, 0.46] |
|  | Italy | 22,945 | 0.29 | [0.26, 0.31] | 0.23 | [0.20, 0.25] | 0.33 | [0.30, 0.35] | 0.27 | [0.24, 0.29] |
|  | Spain | 23,306 | 0.41 | [0.38, 0.44] | 0.36 | [0.33, 0.39] | 0.45 | [0.42, 0.48] | 0.40 | [0.37, 0.43] |
|  | Sweden | 19,547 | 0.18 | [0.15, 0.21] | 0.11 | [0.08, 0.14] | 0.19 | [0.16, 0.22] | 0.13 | [0.10, 0.16] |
|  | Switzerland | 15,203 | 0.36 | [0.33, 0.40] | 0.31 | [0.27, 0.34] | 0.40 | [0.37, 0.44] | 0.34 | [0.31, 0.38] |

Notes: Notes from Table 2 apply, with the exception that none of the specifications include a measure of practice effects as a control.

Appendix Table 6. Odds ratios from logistic regressions for 10-year change in memory impairment, models M1-M4: No adjustment for practice effects

|  |  |  | **M1 - descriptive** | | **M2 - education** | | **M3 - health, demographic** | | **M4 - full adjustment** | |
| --- | --- | --- | --- | --- | --- | --- | --- | --- | --- | --- |
|  |  | **N** | **b** | **95% CI** | **b** | **95% CI** | **b** | **95% CI** | **b** | **95% CI** |
| **Survey** | HRS | 203,400 | 0.84 | [0.81, 0.86] | 0.99 | [0.96, 1.03] | 0.77 | [0.74, 0.80] | 0.93 | [0.89, 0.96] |
|  | ELSA | 64,658 | 0.57 | [0.52, 0.61] | 0.75 | [0.69, 0.81] | 0.54 | [0.49, 0.58] | 0.70 | [0.64, 0.75] |
|  | SHARE | 212,774 | 0.43 | [0.41, 0.45] | 0.50 | [0.48, 0.53] | 0.39 | [0.37, 0.40] | 0.46 | [0.44, 0.48] |
|  | HRS 2004- | 137,548 | 0.88 | [0.83, 0.93] | 1.01 | [0.96, 1.07] | 0.78 | [0.73, 0.82] | 0.91 | [0.85, 0.96] |
|  | ELSA 2004- | 57,419 | 0.66 | [0.60, 0.72] | 0.88 | [0.80, 0.96] | 0.62 | [0.56, 0.68] | 0.81 | [0.74, 0.89] |
|  | |  |  |  |  |  |  |  |  |  |
| **SHARE Countries** | Austria | 17,617 | 0.34 | [0.28, 0.41] | 0.42 | [0.34, 0.50] | 0.35 | [0.28, 0.42] | 0.42 | [0.34, 0.50] |
|  | Belgium | 27,578 | 0.33 | [0.28, 0.37] | 0.41 | [0.36, 0.47] | 0.29 | [0.25, 0.34] | 0.38 | [0.33, 0.44] |
|  | Denmark | 18,316 | 0.70 | [0.58, 0.81] | 0.84 | [0.70, 0.98] | 0.66 | [0.54, 0.77] | 0.79 | [0.65, 0.92] |
|  | France | 22,964 | 0.31 | [0.26, 0.35] | 0.42 | [0.36, 0.47] | 0.29 | [0.25, 0.33] | 0.39 | [0.33, 0.45] |
|  | Germany | 21,454 | 0.48 | [0.40, 0.56] | 0.58 | [0.48, 0.67] | 0.44 | [0.37, 0.52] | 0.54 | [0.44, 0.63] |
|  | Greece | 14,313 | 0.52 | [0.45, 0.60] | 0.59 | [0.50, 0.68] | 0.44 | [0.37, 0.51] | 0.50 | [0.42, 0.58] |
|  | Israel | 9,531 | 0.40 | [0.31, 0.48] | 0.45 | [0.35, 0.54] | 0.33 | [0.26, 0.40] | 0.39 | [0.31, 0.48] |
|  | Italy | 22,945 | 0.43 | [0.37, 0.49] | 0.49 | [0.42, 0.57] | 0.38 | [0.32, 0.43] | 0.44 | [0.37, 0.50] |
|  | Spain | 23,306 | 0.36 | [0.32, 0.40] | 0.39 | [0.35, 0.44] | 0.31 | [0.27, 0.35] | 0.35 | [0.30, 0.39] |
|  | Sweden | 19,547 | 0.61 | [0.53, 0.69] | 0.74 | [0.64, 0.85] | 0.59 | [0.50, 0.67] | 0.70 | [0.60, 0.80] |
|  | Switzerland | 15,203 | 0.44 | [0.36, 0.52] | 0.55 | [0.45, 0.65] | 0.39 | [0.32, 0.47] | 0.50 | [0.40, 0.59] |

Notes: Notes from Table 2 and Appendix Table 4 apply, with the exception that none of the specifications include a measure of practice effects as a control.

Appendix Table 7. Linear time trend coefficients for continuous score, models M1-M4:
Age adjustment by 5-year age groups

|  |  |  | **M1 - descriptive** | | **M2 - education** | | **M3 - health, demographic** | | **M4 - full adjustment** | |
| --- | --- | --- | --- | --- | --- | --- | --- | --- | --- | --- |
|  |  | **N** | **b** | **95% CI** | **b** | **95% CI** | **b** | **95% CI** | **b** | **95% CI** |
| **Survey** | HRS | 203,400 | 0.01 | [0.00, 0.02] | -0.04 | [-0.05, -0.03] | 0.04 | [0.03, 0.04] | -0.02 | [-0.02, -0.01] |
|  | ELSA | 64,658 | 0.15 | [0.13, 0.17] | 0.07 | [0.05, 0.09] | 0.17 | [0.15, 0.19] | 0.09 | [0.08, 0.11] |
|  | SHARE | 212,774 | 0.22 | [0.21, 0.23] | 0.16 | [0.15, 0.17] | 0.25 | [0.24, 0.26] | 0.18 | [0.17, 0.19] |
|  | HRS 2004- | 137,548 | 0.03 | [0.02, 0.04] | -0.01 | [-0.02, -0.00] | 0.07 | [0.06, 0.08] | 0.02 | [0.01, 0.03] |
|  | ELSA 2004- | 57,419 | 0.14 | [0.13, 0.16] | 0.06 | [0.04, 0.08] | 0.16 | [0.15, 0.18] | 0.08 | [0.07, 0.10] |
|  | |  |  |  |  |  |  |  |  |  |
| **SHARE Countries** | Austria | 17,617 | 0.14 | [0.10, 0.18] | 0.10 | [0.06, 0.14] | 0.15 | [0.11, 0.19] | 0.11 | [0.07, 0.15] |
|  | Belgium | 27,578 | 0.31 | [0.28, 0.33] | 0.25 | [0.22, 0.28] | 0.34 | [0.32, 0.37] | 0.28 | [0.25, 0.31] |
|  | Denmark | 18,316 | 0.08 | [0.04, 0.11] | 0.03 | [-0.00, 0.06] | 0.09 | [0.06, 0.13] | 0.05 | [0.02, 0.09] |
|  | France | 22,964 | 0.35 | [0.32, 0.38] | 0.27 | [0.24, 0.30] | 0.36 | [0.33, 0.39] | 0.29 | [0.26, 0.32] |
|  | Germany | 21,454 | 0.20 | [0.17, 0.24] | 0.17 | [0.14, 0.20] | 0.23 | [0.20, 0.27] | 0.19 | [0.16, 0.23] |
|  | Greece | 14,313 | 0.27 | [0.24, 0.30] | 0.22 | [0.19, 0.25] | 0.30 | [0.27, 0.33] | 0.25 | [0.22, 0.28] |
|  | Israel | 9,531 | 0.40 | [0.35, 0.46] | 0.33 | [0.28, 0.38] | 0.44 | [0.38, 0.49] | 0.35 | [0.30, 0.41] |
|  | Italy | 22,945 | 0.26 | [0.23, 0.29] | 0.20 | [0.17, 0.23] | 0.30 | [0.26, 0.33] | 0.24 | [0.21, 0.27] |
|  | Spain | 23,306 | 0.35 | [0.32, 0.38] | 0.30 | [0.26, 0.33] | 0.38 | [0.34, 0.41] | 0.33 | [0.30, 0.36] |
|  | Sweden | 19,547 | 0.11 | [0.08, 0.14] | 0.04 | [0.01, 0.07] | 0.12 | [0.09, 0.15] | 0.05 | [0.02, 0.09] |
|  | Switzerland | 15,203 | 0.28 | [0.24, 0.32] | 0.21 | [0.17, 0.25] | 0.31 | [0.27, 0.36] | 0.25 | [0.21, 0.29] |

Notes: Notes from Table 2 apply, with the exception that age now enters the regression as a categorical variable (5-year age groups) and that the model is estimated with a random intercept only.

Appendix Table 8. Linear time trend coefficients for continuous score, models M1-M4:
Practice effect measure is categorical variable.

|  |  |  | **M1 - descriptive** | | **M2 - education** | | **M3 - health, demographic** | | **M4 - full adjustment** | |
| --- | --- | --- | --- | --- | --- | --- | --- | --- | --- | --- |
|  |  | **N** | **b** | **95% CI** | **b** | **95% CI** | **b** | **95% CI** | **b** | **95% CI** |
| **Survey** | HRS | 203,400 | -0.01 | [-0.02, -0.00] | -0.06 | [-0.07, -0.05] | 0.01 | [0.00, 0.02] | -0.04 | [-0.05, -0.03] |
|  | ELSA | 64,658 | 0.08 | [0.05, 0.11] | 0.00 | [-0.03, 0.02] | 0.10 | [0.07, 0.13] | 0.02 | [-0.01, 0.04] |
|  | SHARE | 212,774 | 0.20 | [0.19, 0.22] | 0.14 | [0.12, 0.15] | 0.22 | [0.21, 0.23] | 0.15 | [0.14, 0.16] |
|  | HRS 2004- | 137,548 | 0.02 | [0.01, 0.04] | -0.02 | [-0.03, -0.01] | 0.06 | [0.04, 0.07] | 0.01 | [-0.00, 0.02] |
|  | ELSA 2004- | 57,419 | 0.06 | [0.03, 0.09] | -0.03 | [-0.06, 0.00] | 0.07 | [0.04, 0.10] | -0.01 | [-0.04, 0.02] |
|  | |  |  |  |  |  |  |  |  |  |
| **SHARE Countries** | Austria | 17,617 | 0.25 | [0.19, 0.32] | 0.19 | [0.13, 0.25] | 0.24 | [0.18, 0.30] | 0.18 | [0.12, 0.24] |
|  | Belgium | 27,578 | 0.29 | [0.25, 0.33] | 0.21 | [0.18, 0.25] | 0.33 | [0.29, 0.37] | 0.24 | [0.20, 0.28] |
|  | Denmark | 18,316 | 0.09 | [0.04, 0.14] | 0.05 | [0.00, 0.10] | 0.08 | [0.04, 0.13] | 0.05 | [0.00, 0.10] |
|  | France | 22,964 | 0.35 | [0.31, 0.40] | 0.30 | [0.25, 0.34] | 0.35 | [0.30, 0.39] | 0.29 | [0.25, 0.34] |
|  | Germany | 21,454 | 0.16 | [0.11, 0.20] | 0.14 | [0.09, 0.18] | 0.18 | [0.14, 0.23] | 0.16 | [0.11, 0.20] |
|  | Greece | 14,313 | 0.32 | [0.28, 0.35] | 0.27 | [0.24, 0.31] | 0.35 | [0.31, 0.39] | 0.31 | [0.27, 0.34] |
|  | Israel | 9,531 | 0.40 | [0.33, 0.48] | 0.25 | [0.18, 0.33] | 0.42 | [0.34, 0.50] | 0.28 | [0.20, 0.36] |
|  | Italy | 22,945 | 0.31 | [0.26, 0.35] | 0.25 | [0.21, 0.29] | 0.31 | [0.27, 0.35] | 0.26 | [0.22, 0.30] |
|  | Spain | 23,306 | 0.38 | [0.34, 0.42] | 0.32 | [0.28, 0.36] | 0.37 | [0.33, 0.41] | 0.33 | [0.29, 0.37] |
|  | Sweden | 19,547 | 0.06 | [0.01, 0.10] | -0.03 | [-0.07, 0.02] | 0.05 | [0.01, 0.10] | -0.02 | [-0.06, 0.02] |
|  | Switzerland | 15,203 | 0.32 | [0.26, 0.39] | 0.19 | [0.13, 0.26] | 0.35 | [0.28, 0.42] | 0.23 | [0.16, 0.29] |

Notes: Notes from Table 2 apply, with the exception that practice effects are measured differentlyInstead of a binary indicator for the first test occasion, a categorical variable with five bins is used (test occasions 1, 2, 3-4, 5-7, and 8+).

Appendix Table 9. Linear time trend coefficients for continuous score, models M1-M4: Samples restricted to first-time test takers.

|  |  |  | **M1 - descriptive** | | **M2 - education** | | **M3 - health, demographic** | | **M4 - full adjustment** | |
| --- | --- | --- | --- | --- | --- | --- | --- | --- | --- | --- |
|  |  | **N** | **b** | **95% CI** | **b** | **95% CI** | **b** | **95% CI** | **b** | **95% CI** |
| **Survey** | HRS | 34,258 | -0.05 | [-0.07, -0.04] | -0.11 | [-0.12, -0.09] | -0.04 | [-0.06, -0.03] | -0.10 | [-0.11, -0.08] |
|  | ELSA | 10,822 | 0.22 | [0.16, 0.27] | 0.13 | [0.07, 0.18] | 0.22 | [0.17, 0.28] | 0.15 | [0.09, 0.20] |
|  | SHARE | 63,952 | 0.27 | [0.25, 0.29] | 0.20 | [0.19, 0.22] | 0.28 | [0.26, 0.30] | 0.21 | [0.20, 0.23] |
|  | HRS 2004- | 13,518 | -0.03 | [-0.06, 0.01] | -0.04 | [-0.08, -0.01] | -0.01 | [-0.04, 0.03] | -0.03 | [-0.06, 0.00] |
|  | ELSA 2004- | 3,583 | 0.28 | [0.12, 0.43] | 0.20 | [0.06, 0.35] | 0.28 | [0.13, 0.43] | 0.21 | [0.07, 0.35] |
|  | |  |  |  |  |  |  |  |  |  |
| **SHARE Countries** | Austria | 5,365 | 0.39 | [0.31, 0.47] | 0.31 | [0.23, 0.39] | 0.36 | [0.27, 0.44] | 0.29 | [0.21, 0.37] |
|  | Belgium | 8,159 | 0.39 | [0.34, 0.44] | 0.30 | [0.25, 0.35] | 0.42 | [0.37, 0.47] | 0.33 | [0.28, 0.38] |
|  | Denmark | 4,966 | 0.13 | [0.07, 0.19] | 0.11 | [0.04, 0.17] | 0.10 | [0.04, 0.16] | 0.08 | [0.02, 0.14] |
|  | France | 6,582 | 0.44 | [0.38, 0.51] | 0.41 | [0.35, 0.48] | 0.42 | [0.35, 0.48] | 0.39 | [0.33, 0.45] |
|  | Germany | 6,900 | 0.16 | [0.11, 0.22] | 0.14 | [0.09, 0.20] | 0.21 | [0.15, 0.26] | 0.18 | [0.13, 0.24] |
|  | Greece | 5,555 | 0.33 | [0.29, 0.38] | 0.28 | [0.23, 0.32] | 0.36 | [0.31, 0.40] | 0.30 | [0.26, 0.35] |
|  | Israel | 2,693 | 0.34 | [0.23, 0.45] | 0.11 | [0.01, 0.22] | 0.33 | [0.22, 0.44] | 0.12 | [0.01, 0.23] |
|  | Italy | 7,133 | 0.36 | [0.31, 0.41] | 0.31 | [0.26, 0.36] | 0.34 | [0.29, 0.39] | 0.31 | [0.26, 0.36] |
|  | Spain | 7,191 | 0.45 | [0.40, 0.50] | 0.39 | [0.34, 0.44] | 0.43 | [0.37, 0.48] | 0.39 | [0.34, 0.44] |
|  | Sweden | 5,372 | 0.10 | [0.04, 0.15] | 0.01 | [-0.05, 0.07] | 0.07 | [0.02, 0.13] | 0.00 | [-0.06, 0.06] |
|  | Switzerland | 4,036 | 0.43 | [0.33, 0.52] | 0.24 | [0.15, 0.34] | 0.44 | [0.34, 0.53] | 0.26 | [0.16, 0.36] |

Notes: Based on regressions restricted to samples of first-time test takers. Numbers for HRS and ELSA are not shown, since only SHARE has a suitable sample refreshment scheme to support this particular specification. The estimation methodology used is ordinary least squares. Since there is only one observation per subject, panel data estimators like those from random intercept models are not applicable here. Other than that, notes from Table 2 apply.

Appendix Table 10. Comparison of main logistic HRS regressions to Hale et al. (2020)

|  | **Adjustment: PE** | | **Adjustment: PE + education** | |
| --- | --- | --- | --- | --- |
| Original study results |  |  |  |  |
| Hale et al. 2020 (last wave: 2014) | 1.29 | [1.16, 1.44] | 1.54 | [1.39, 1.70] |
| Current study (CS-ME) | 0.86 | [0.83, 0.90] | 1.03 | [0.99, 1.06] |
| Current study, no mixed effects (CS) | 0.84 | [0.82, 0.86] | 0.95 | [0.93, 0.98] |
| Variations of current study |  |  |  |  |
| CS, but PE adj. is 5-cat. | 1.17 | [1.14, 1.20] | 1.30 | [1.27, 1.34] |
| CS, but without common sample restriction | 0.92 | [0.90, 0.94] | 1.03 | [1.01, 1.06] |
| CS, but using data up to 2014 only | 0.87 | [0.84, 0.90] | 1.00 | [0.97, 1.04] |
| CS, but using complex survey design | 0.80 | [0.76, 0.84] | 0.91 | [0.87, 0.96] |
| CS, but impairment is 0-6 of 27 cog score | 0.82 | [0.79, 0.85] | 1.00 | [0.97, 1.04] |
| CS, but using proxy interviews | 0.92 | [0.90, 0.94] | 1.03 | [1.01, 1.05] |
| CS, but using 27 cog score and proxies | 0.98 | [0.95, 1.01] | 1.15 | [1.12, 1.19] |
| CS, but using the HRS education variable | 0.84 | [0.82, 0.86] | 0.96 | [0.94, 0.99] |
| Hale at al specification |  |  |  |  |
| CS, with all of the above (last wave: 2018) | 1.25 | [1.17, 1.34] | 1.47 | [1.38, 1.57] |

Notes: Comparison of current study (CS-ME) specifications with results from Hale et al. 2020 (henceforth: HEA) (1), as well as various modifications of the baseline specifications of the current study. Values shown are odds ratios for memory impairment for 10-year trend coefficients along with corresponding 95% confidence intervals. Columns "Adjustment: PE" and "Adjustment: PE + education" correspond to Model 1 and 2 of the current study, respectively. The first table section contrasts the results of HEA and of the current study. HEA do not use mixed effects as does CS, so for direct comparability line 3 of the first table section presents results (CS) that do not feature random intercept and random age slope. The second table section adapts the specifications of the current study towards the ones in HEA in order to investigate where differences in the estimates stem from. It does so by varying one specification setting at a time. Exception to the principle: just varying a single specification is the second to last line of the middle table section, which applies the changes of the previous two lines simultaneously (6-out-of-27 points dementia cutoff, proxy interviews) in order to apply a consistent definition of dementia across interview types (self-respondent or proxy). The bottom table section applies all specification modifications at once, making the regressions of the current study fully compatible with the ones in HEA, except for the sample span, which ends in 2014 for HEA and 2018 for the current study.

Appendix Table 11. Main linear HRS regressions exclusive of observations with imputed word recall scores

|  | **M1 - descriptive** | | | **M2 - education** | | **M3 - health, demographic** | | **M4 - full adjustment** | |
| --- | --- | --- | --- | --- | --- | --- | --- | --- | --- |
|  | **N** | **b** | **95% CI** | **b** | **95% CI** | **b** | **95% CI** | **b** | **95% CI** |
| Imputations |  |  |  |  |  |  |  |  |  |
| Yes | 203,400 | 0.02 | [0.01, 0.02] | -0.04 | [-0.04, -0.03] | 0.04 | [0.04, 0.05] | -0.01 | [-0.02, -0.01] |
| No | 191,880 | 0.00 | [-0.01, 0.01] | -0.05 | [-0.06, -0.05] | 0.02 | [0.02, 0.03] | -0.03 | [-0.04, -0.02] |

Notes: Comparing standardized 10-year time trend coefficients in cognition for baseline linear HRS regressions, models M1-M4, with corresponding regressions whose samples exclude imputed word recall scores. Notes from Table 2 apply.

Appendix Table 12. Comparison of ELSA regressions when sample is not restricted by the availability of BMI data

|  | **M1 - descriptive** | | | | **M2 - education** | | | |
| --- | --- | --- | --- | --- | --- | --- | --- | --- |
|  | **BMI-restricted** | | **Unrestricted** | | **BMI-restricted** | | **Unrestricted** | |
|  | **b** | **95% CI** | **b** | **95% CI** | **b** | **95% CI** | **b** | **95% CI** |
| Continuous score |  |  |  |  |  |  |  |  |
| Baseline | 0.18 | [0.16, 0.20] | 0.18 | [0.16, 0.19] | 0.09 | [0.07, 0.11] | 0.09 | [0.07, 0.10] |
| No adj. for prior testing | 0.22 | [0.21, 0.24] | 0.22 | [0.21, 0.23] | 0.13 | [0.11, 0.15] | 0.13 | [0.11, 0.14] |
| Test adj.: 1/2/3-4/5-7/8+ tests | 0.08 | [0.05, 0.11] | 0.08 | [0.06, 0.10] | 0.00 | [-0.03, 0.02] | 0.00 | [-0.02, 0.02] |
| Logistic regression |  |  |  |  |  |  |  |  |
| Baseline | 0.67 | [0.61, 0.73] | 0.62 | [0.57, 0.67] | 0.89 | [0.81, 0.97] | 0.83 | [0.76, 0.89] |
| No adj. for prior testing | 0.57 | [0.52, 0.61] | 0.50 | [0.46, 0.53] | 0.75 | [0.69, 0.81] | 0.67 | [0.63, 0.72] |

Notes: 10-Year trend coefficients and corresponding 95% confidence intervals for ELSA data comparing samples that are restricted by data availability of BMI versus samples that are not restricted in that respect. Comparisons are performed for models M1 and M2, which do not contain BMI as a right-hand side variable. Continuous score regressions are compared for the baseline specification (Table 2), for specifications that do not control for the number of prior test occasions (Appendix Table 5), and for specifications whose control variable for the number of prior tests consists solely of a binary indicator for the first test occasion (Appendix Table 8). Logistic regressions for binary memory impairment are compared for the baseline specification (Appendix Table 4) and for specifications that do not control for the number of prior test occasions (Appendix Table 6). Dropping the sample restriction of BMI availability increases the sample from 64,658 observations to 76,330 observations for all specifications.

Appendix Table 13. Linear time trend coefficients for continuous score, models M1-M4: Dependent variable is immediate recall score

|  |  |  | **M1 - descriptive** | | **M2 - education** | | **M3 - health, demographic** | | **M4 - full adjustment** | |
| --- | --- | --- | --- | --- | --- | --- | --- | --- | --- | --- |
|  |  | **N** | **b** | **95% CI** | **b** | **95% CI** | **b** | **95% CI** | **b** | **95% CI** |
| **Survey** | HRS | 203,400 | 0.00 | [-0.01, 0.01] | -0.05 | [-0.06, -0.05] | 0.02 | [0.02, 0.03] | -0.03 | [-0.04, -0.02] |
|  | ELSA | 64,658 | 0.18 | [0.16, 0.20] | 0.10 | [0.08, 0.11] | 0.20 | [0.18, 0.21] | 0.11 | [0.10, 0.13] |
|  | SHARE | 212,774 | 0.23 | [0.22, 0.24] | 0.16 | [0.15, 0.17] | 0.25 | [0.24, 0.26] | 0.18 | [0.17, 0.19] |
|  | HRS 2004- | 137,548 | 0.03 | [0.02, 0.04] | -0.02 | [-0.03, -0.01] | 0.06 | [0.05, 0.07] | 0.02 | [0.01, 0.03] |
|  | ELSA 2004- | 57,419 | 0.18 | [0.16, 0.20] | 0.09 | [0.07, 0.11] | 0.19 | [0.17, 0.21] | 0.11 | [0.09, 0.13] |
|  | |  |  |  |  |  |  |  |  |  |
| **SHARE Countries** | Austria | 17,617 | 0.21 | [0.17, 0.25] | 0.16 | [0.12, 0.20] | 0.20 | [0.16, 0.24] | 0.15 | [0.11, 0.20] |
|  | Belgium | 27,578 | 0.28 | [0.25, 0.31] | 0.22 | [0.19, 0.25] | 0.31 | [0.28, 0.34] | 0.25 | [0.22, 0.27] |
|  | Denmark | 18,316 | 0.09 | [0.05, 0.13] | 0.05 | [0.01, 0.08] | 0.10 | [0.06, 0.13] | 0.06 | [0.02, 0.09] |
|  | France | 22,964 | 0.34 | [0.31, 0.37] | 0.27 | [0.24, 0.30] | 0.35 | [0.32, 0.38] | 0.28 | [0.25, 0.31] |
|  | Germany | 21,454 | 0.15 | [0.11, 0.18] | 0.12 | [0.08, 0.15] | 0.17 | [0.13, 0.20] | 0.13 | [0.10, 0.17] |
|  | Greece | 14,313 | 0.28 | [0.25, 0.31] | 0.24 | [0.21, 0.27] | 0.31 | [0.28, 0.34] | 0.27 | [0.24, 0.30] |
|  | Israel | 9,531 | 0.38 | [0.32, 0.44] | 0.30 | [0.24, 0.35] | 0.41 | [0.35, 0.46] | 0.32 | [0.26, 0.37] |
|  | Italy | 22,945 | 0.29 | [0.25, 0.32] | 0.23 | [0.20, 0.26] | 0.32 | [0.28, 0.35] | 0.26 | [0.23, 0.29] |
|  | Spain | 23,306 | 0.39 | [0.36, 0.42] | 0.34 | [0.30, 0.37] | 0.41 | [0.38, 0.44] | 0.36 | [0.33, 0.40] |
|  | Sweden | 19,547 | 0.08 | [0.05, 0.12] | 0.01 | [-0.02, 0.04] | 0.09 | [0.05, 0.12] | 0.02 | [-0.02, 0.05] |
|  | Switzerland | 15,203 | 0.24 | [0.20, 0.29] | 0.17 | [0.12, 0.21] | 0.27 | [0.22, 0.31] | 0.19 | [0.15, 0.24] |

Notes: Notes from Table 2 apply, with the exception that the dependent variable is not the sum of immediate and delayed word recall scores (range 0-20), but based on immediate recall only (range 0-10).

Appendix Table 14. Linear time trend coefficients for continuous score, models M1-M4: Dependent variable is delayed recall score

|  |  |  | **M1 - descriptive** | | **M2 - education** | | **M3 - health, demographic** | | **M4 - full adjustment** | |
| --- | --- | --- | --- | --- | --- | --- | --- | --- | --- | --- |
|  |  | **N** | **b** | **95% CI** | **b** | **95% CI** | **b** | **95% CI** | **b** | **95% CI** |
| **Survey** | HRS | 203,400 | 0.03 | [0.02, 0.04] | -0.02 | [-0.02, -0.01] | 0.05 | [0.05, 0.06] | 0.00 | [-0.00, 0.01] |
|  | ELSA | 64,658 | 0.17 | [0.15, 0.18] | 0.08 | [0.06, 0.10] | 0.18 | [0.16, 0.20] | 0.10 | [0.08, 0.12] |
|  | SHARE | 212,774 | 0.22 | [0.21, 0.23] | 0.16 | [0.15, 0.17] | 0.25 | [0.24, 0.26] | 0.18 | [0.17, 0.19] |
|  | HRS 2004- | 137,548 | 0.04 | [0.03, 0.05] | 0.00 | [-0.01, 0.01] | 0.08 | [0.07, 0.09] | 0.03 | [0.02, 0.04] |
|  | ELSA 2004- | 57,419 | 0.15 | [0.14, 0.17] | 0.07 | [0.05, 0.09] | 0.17 | [0.15, 0.19] | 0.09 | [0.07, 0.10] |
|  | |  |  |  |  |  |  |  |  |  |
| **SHARE Countries** | Austria | 17,617 | 0.14 | [0.10, 0.18] | 0.10 | [0.06, 0.14] | 0.15 | [0.11, 0.19] | 0.11 | [0.07, 0.15] |
|  | Belgium | 27,578 | 0.33 | [0.30, 0.36] | 0.27 | [0.24, 0.30] | 0.36 | [0.33, 0.39] | 0.30 | [0.27, 0.32] |
|  | Denmark | 18,316 | 0.08 | [0.05, 0.12] | 0.04 | [0.01, 0.08] | 0.10 | [0.06, 0.13] | 0.06 | [0.02, 0.09] |
|  | France | 22,964 | 0.34 | [0.31, 0.37] | 0.27 | [0.24, 0.30] | 0.35 | [0.32, 0.38] | 0.28 | [0.25, 0.31] |
|  | Germany | 21,454 | 0.23 | [0.20, 0.27] | 0.20 | [0.17, 0.24] | 0.26 | [0.23, 0.30] | 0.23 | [0.19, 0.26] |
|  | Greece | 14,313 | 0.24 | [0.21, 0.27] | 0.20 | [0.16, 0.23] | 0.27 | [0.23, 0.30] | 0.22 | [0.19, 0.26] |
|  | Israel |  |  |  | 0.33 | [0.28, 0.39] |  |  | 0.35 | [0.30, 0.41] |
|  | Italy | 22,945 | 0.22 | [0.19, 0.26] | 0.17 | [0.14, 0.21] | 0.25 | [0.22, 0.28] | 0.20 | [0.17, 0.23] |
|  | Spain | 23,306 | 0.30 | [0.26, 0.33] | 0.25 | [0.22, 0.28] | 0.32 | [0.29, 0.35] | 0.28 | [0.24, 0.31] |
|  | Sweden | 19,547 | 0.14 | [0.11, 0.17] | 0.08 | [0.05, 0.11] | 0.15 | [0.11, 0.18] | 0.09 | [0.06, 0.12] |
|  | Switzerland | 15,203 | 0.32 | [0.28, 0.36] | 0.26 | [0.22, 0.30] | 0.35 | [0.31, 0.40] | 0.29 | [0.25, 0.34] |

Notes: Notes from Table 2 apply, with the exception that the dependent variable is not the sum of immediate and delayed word recall scores (range 0-20), but based on delayed recall only (range 0-10).

Appendix Table 15. Linear time trend coefficients for continuous score, models M1-M4: Sample excluding nursing home residents

|  |  |  | **M1 - descriptive** | | **M2 - education** | | **M3 - health, demographic** | | **M4 - full adjustment** | |
| --- | --- | --- | --- | --- | --- | --- | --- | --- | --- | --- |
|  |  | **N** | **b** | **95% CI** | **b** | **95% CI** | **b** | **95% CI** | **b** | **95% CI** |
| **Survey** | HRS | 201,708 | 0.02 | [0.01, 0.03] | -0.04 | [-0.04, -0.03] | 0.04 | [0.04, 0.05] | -0.01 | [-0.02, -0.01] |
|  | ELSA | 64,608 | 0.18 | [0.17, 0.20] | 0.09 | [0.07, 0.11] | 0.20 | [0.18, 0.22] | 0.11 | [0.09, 0.13] |
|  | SHARE | 211,651 | 0.24 | [0.23, 0.25] | 0.17 | [0.16, 0.18] | 0.27 | [0.26, 0.28] | 0.20 | [0.19, 0.21] |
|  | HRS 2004- | 136,311 | 0.04 | [0.03, 0.05] | -0.01 | [-0.02, 0.00] | 0.07 | [0.06, 0.08] | 0.02 | [0.01, 0.03] |
|  | ELSA 2004- | 57,369 | 0.17 | [0.15, 0.19] | 0.08 | [0.06, 0.10] | 0.19 | [0.17, 0.21] | 0.10 | [0.08, 0.12] |
|  | |  |  |  |  |  |  |  |  |  |
| **SHARE Countries** | Austria | 17,515 | 0.18 | [0.14, 0.22] | 0.13 | [0.09, 0.17] | 0.18 | [0.14, 0.22] | 0.14 | [0.09, 0.18] |
|  | Belgium | 27,321 | 0.34 | [0.31, 0.36] | 0.27 | [0.25, 0.30] | 0.37 | [0.34, 0.40] | 0.30 | [0.27, 0.33] |
|  | Denmark | 18,201 | 0.10 | [0.06, 0.13] | 0.05 | [0.02, 0.09] | 0.11 | [0.08, 0.14] | 0.07 | [0.04, 0.10] |
|  | France | 22,841 | 0.37 | [0.34, 0.40] | 0.29 | [0.26, 0.32] | 0.38 | [0.35, 0.41] | 0.30 | [0.27, 0.33] |
|  | Germany | 21,330 | 0.22 | [0.18, 0.25] | 0.18 | [0.15, 0.22] | 0.24 | [0.21, 0.28] | 0.21 | [0.17, 0.24] |
|  | Greece | 14,308 | 0.27 | [0.24, 0.30] | 0.23 | [0.20, 0.26] | 0.30 | [0.27, 0.33] | 0.26 | [0.23, 0.29] |
|  | Israel | 9,477 | 0.43 | [0.37, 0.48] | 0.34 | [0.29, 0.40] | 0.45 | [0.40, 0.51] | 0.36 | [0.31, 0.42] |
|  | Italy | 22,906 | 0.27 | [0.24, 0.30] | 0.22 | [0.19, 0.25] | 0.30 | [0.27, 0.33] | 0.25 | [0.22, 0.28] |
|  | Spain | 23,223 | 0.37 | [0.34, 0.40] | 0.31 | [0.28, 0.35] | 0.39 | [0.36, 0.42] | 0.34 | [0.31, 0.37] |
|  | Sweden | 19,444 | 0.13 | [0.09, 0.16] | 0.06 | [0.02, 0.09] | 0.13 | [0.10, 0.17] | 0.07 | [0.03, 0.10] |
|  | Switzerland | 15,085 | 0.31 | [0.27, 0.36] | 0.24 | [0.20, 0.28] | 0.35 | [0.30, 0.39] | 0.28 | [0.23, 0.32] |

Notes: Notes from Table 2 apply, with the exception that the sample excludes observations for nursing home residents.

Appendix Table 16. Linear time trend coefficients for continuous score, models M1-M4: Sample restricted to ages 60 and over

|  |  |  | **M1 - descriptive** | | **M2 - education** | | **M3 - health, demographic** | | **M4 - full adjustment** | |
| --- | --- | --- | --- | --- | --- | --- | --- | --- | --- | --- |
|  |  | **N** | **b** | **95% CI** | **b** | **95% CI** | **b** | **95% CI** | **b** | **95% CI** |
| **Survey** | HRS | 145,941 | 0.05 | [0.04, 0.06] | -0.01 | [-0.02, -0.00] | 0.08 | [0.07, 0.09] | 0.02 | [0.01, 0.02] |
|  | ELSA | 48,408 | 0.19 | [0.17, 0.21] | 0.09 | [0.07, 0.11] | 0.20 | [0.18, 0.22] | 0.11 | [0.09, 0.13] |
|  | SHARE | 156,139 | 0.23 | [0.22, 0.24] | 0.15 | [0.14, 0.17] | 0.27 | [0.25, 0.28] | 0.18 | [0.17, 0.20] |
|  | HRS 2004- | 97,498 | 0.05 | [0.04, 0.07] | 0.00 | [-0.01, 0.01] | 0.10 | [0.08, 0.11] | 0.04 | [0.03, 0.05] |
|  | ELSA 2004- | 44,118 | 0.18 | [0.16, 0.20] | 0.09 | [0.07, 0.11] | 0.20 | [0.18, 0.22] | 0.11 | [0.09, 0.13] |
|  | |  |  |  |  |  |  |  |  |  |
| **SHARE Countries** | Austria | 13,637 | 0.18 | [0.13, 0.23] | 0.12 | [0.08, 0.17] | 0.19 | [0.14, 0.23] | 0.13 | [0.09, 0.18] |
|  | Belgium | 18,524 | 0.31 | [0.27, 0.34] | 0.24 | [0.20, 0.27] | 0.34 | [0.30, 0.37] | 0.26 | [0.23, 0.30] |
|  | Denmark | 12,282 | 0.08 | [0.04, 0.12] | 0.03 | [-0.02, 0.07] | 0.10 | [0.05, 0.14] | 0.05 | [0.00, 0.09] |
|  | France | 16,490 | 0.33 | [0.29, 0.37] | 0.25 | [0.22, 0.29] | 0.34 | [0.31, 0.38] | 0.27 | [0.23, 0.30] |
|  | Germany | 15,251 | 0.19 | [0.15, 0.23] | 0.14 | [0.10, 0.18] | 0.21 | [0.17, 0.26] | 0.16 | [0.12, 0.20] |
|  | Greece | 10,136 | 0.26 | [0.23, 0.30] | 0.22 | [0.18, 0.26] | 0.30 | [0.27, 0.34] | 0.26 | [0.22, 0.30] |
|  | Israel | 7,384 | 0.44 | [0.38, 0.50] | 0.37 | [0.31, 0.43] | 0.48 | [0.41, 0.54] | 0.40 | [0.34, 0.46] |
|  | Italy | 16,960 | 0.28 | [0.25, 0.32] | 0.23 | [0.19, 0.26] | 0.32 | [0.29, 0.36] | 0.27 | [0.23, 0.30] |
|  | Spain | 17,943 | 0.36 | [0.32, 0.40] | 0.31 | [0.27, 0.35] | 0.39 | [0.36, 0.43] | 0.35 | [0.31, 0.38] |
|  | Sweden | 16,197 | 0.11 | [0.08, 0.15] | 0.04 | [0.00, 0.08] | 0.12 | [0.09, 0.16] | 0.06 | [0.02, 0.09] |
|  | Switzerland | 11,335 | 0.29 | [0.24, 0.33] | 0.22 | [0.17, 0.26] | 0.32 | [0.27, 0.37] | 0.25 | [0.20, 0.30] |

Notes: Notes from Table 2 apply, with the exception that the sample is restricted to subjects of ages 60 and over at the time of the interview.

Appendix Table 17. Linear time trend coefficients for continuous score, models M1-M4: Least squares regression

|  |  |  | **M1 - descriptive** | | **M2 - education** | | **M3 - health, demographic** | | **M4 - full adjustment** | |
| --- | --- | --- | --- | --- | --- | --- | --- | --- | --- | --- |
|  |  | **N** | **b** | **95% CI** | **b** | **95% CI** | **b** | **95% CI** | **b** | **95% CI** |
| **Survey** | HRS | 203,400 | 0.04 | [0.03, 0.05] | -0.02 | [-0.03, -0.01] | 0.06 | [0.05, 0.06] | 0.00 | [-0.01, 0.01] |
|  | ELSA | 64,658 | 0.23 | [0.21, 0.25] | 0.13 | [0.11, 0.15] | 0.23 | [0.21, 0.25] | 0.14 | [0.12, 0.16] |
|  | SHARE | 212,774 | 0.28 | [0.26, 0.29] | 0.19 | [0.18, 0.20] | 0.30 | [0.28, 0.31] | 0.21 | [0.20, 0.22] |
|  | HRS 2004- | 137,548 | 0.07 | [0.06, 0.08] | 0.02 | [0.01, 0.03] | 0.10 | [0.09, 0.11] | 0.05 | [0.04, 0.06] |
|  | ELSA 2004- | 57,419 | 0.23 | [0.21, 0.25] | 0.13 | [0.11, 0.15] | 0.23 | [0.21, 0.25] | 0.14 | [0.12, 0.16] |
|  | |  |  |  |  |  |  |  |  |  |
| **SHARE Countries** | Austria | 17,617 | 0.36 | [0.31, 0.41] | 0.28 | [0.23, 0.33] | 0.32 | [0.27, 0.36] | 0.25 | [0.21, 0.30] |
|  | Belgium | 27,578 | 0.37 | [0.34, 0.40] | 0.29 | [0.26, 0.32] | 0.40 | [0.37, 0.43] | 0.31 | [0.28, 0.34] |
|  | Denmark | 18,316 | 0.11 | [0.07, 0.15] | 0.07 | [0.03, 0.11] | 0.10 | [0.06, 0.14] | 0.06 | [0.02, 0.10] |
|  | France | 22,964 | 0.39 | [0.35, 0.42] | 0.32 | [0.28, 0.35] | 0.38 | [0.35, 0.42] | 0.31 | [0.28, 0.35] |
|  | Germany | 21,454 | 0.23 | [0.19, 0.27] | 0.20 | [0.16, 0.24] | 0.26 | [0.22, 0.30] | 0.22 | [0.18, 0.26] |
|  | Greece | 14,313 | 0.29 | [0.25, 0.32] | 0.24 | [0.21, 0.28] | 0.32 | [0.29, 0.35] | 0.28 | [0.25, 0.31] |
|  | Israel | 9,531 | 0.43 | [0.37, 0.49] | 0.31 | [0.25, 0.37] | 0.45 | [0.39, 0.51] | 0.34 | [0.28, 0.40] |
|  | Italy | 22,945 | 0.29 | [0.26, 0.33] | 0.24 | [0.20, 0.27] | 0.31 | [0.27, 0.34] | 0.26 | [0.23, 0.29] |
|  | Spain | 23,306 | 0.41 | [0.37, 0.44] | 0.34 | [0.31, 0.38] | 0.41 | [0.37, 0.44] | 0.36 | [0.32, 0.39] |
|  | Sweden | 19,547 | 0.15 | [0.11, 0.19] | 0.06 | [0.03, 0.10] | 0.14 | [0.10, 0.18] | 0.06 | [0.02, 0.10] |
|  | Switzerland | 15,203 | 0.37 | [0.32, 0.42] | 0.25 | [0.20, 0.30] | 0.39 | [0.34, 0.44] | 0.28 | [0.23, 0.33] |

Notes: Notes from Table 2 apply, with the exception that the model does not have random intercepts. It is estimated by OLS with standard errors clustered by subject.

Appendix Table 18. Linear time trend coefficients for continuous score, models M1-M4: No random age slopes

|  |  |  | **M1 - descriptive** | | **M2 - education** | | **M3 - health, demographic** | | **M4 - full adjustment** | |
| --- | --- | --- | --- | --- | --- | --- | --- | --- | --- | --- |
|  |  | **N** | **b** | **95% CI** | **b** | **95% CI** | **b** | **95% CI** | **b** | **95% CI** |
| **Survey** | HRS | 203,400 | 0.02 | [0.02, 0.03] | -0.03 | [-0.04, -0.02] | 0.05 | [0.04, 0.05] | -0.01 | [-0.02, -0.00] |
|  | ELSA | 64,658 | 0.19 | [0.17, 0.21] | 0.10 | [0.08, 0.12] | 0.20 | [0.19, 0.22] | 0.12 | [0.10, 0.14] |
|  | SHARE | 212,774 | 0.24 | [0.23, 0.25] | 0.17 | [0.16, 0.18] | 0.27 | [0.26, 0.28] | 0.19 | [0.18, 0.20] |
|  | HRS 2004- | 137,548 | 0.04 | [0.03, 0.05] | 0.00 | [-0.01, 0.01] | 0.08 | [0.07, 0.09] | 0.03 | [0.02, 0.04] |
|  | ELSA 2004- | 57,419 | 0.18 | [0.16, 0.20] | 0.09 | [0.07, 0.11] | 0.19 | [0.18, 0.21] | 0.11 | [0.09, 0.12] |
|  | |  |  |  |  |  |  |  |  |  |
| **SHARE Countries** | Austria | 17,617 | 0.17 | [0.13, 0.21] | 0.12 | [0.08, 0.16] | 0.17 | [0.13, 0.21] | 0.12 | [0.08, 0.16] |
|  | Belgium | 27,578 | 0.33 | [0.30, 0.35] | 0.27 | [0.24, 0.29] | 0.36 | [0.33, 0.39] | 0.29 | [0.27, 0.32] |
|  | Denmark | 18,316 | 0.09 | [0.06, 0.13] | 0.05 | [0.02, 0.08] | 0.11 | [0.08, 0.14] | 0.07 | [0.03, 0.10] |
|  | France | 22,964 | 0.36 | [0.34, 0.39] | 0.29 | [0.26, 0.32] | 0.38 | [0.35, 0.40] | 0.30 | [0.27, 0.33] |
|  | Germany | 21,454 | 0.22 | [0.19, 0.25] | 0.19 | [0.15, 0.22] | 0.25 | [0.21, 0.28] | 0.21 | [0.17, 0.24] |
|  | Greece | 14,313 | 0.27 | [0.24, 0.30] | 0.23 | [0.20, 0.26] | 0.30 | [0.27, 0.33] | 0.26 | [0.23, 0.29] |
|  | Israel | 9,531 | 0.42 | [0.36, 0.47] | 0.34 | [0.29, 0.39] | 0.45 | [0.39, 0.50] | 0.36 | [0.31, 0.42] |
|  | Italy | 22,945 | 0.27 | [0.24, 0.31] | 0.22 | [0.19, 0.25] | 0.31 | [0.28, 0.34] | 0.25 | [0.22, 0.28] |
|  | Spain | 23,306 | 0.36 | [0.33, 0.39] | 0.31 | [0.28, 0.34] | 0.39 | [0.36, 0.42] | 0.34 | [0.31, 0.37] |
|  | Sweden | 19,547 | 0.13 | [0.10, 0.16] | 0.06 | [0.03, 0.09] | 0.14 | [0.10, 0.17] | 0.07 | [0.04, 0.10] |
|  | Switzerland | 15,203 | 0.30 | [0.26, 0.34] | 0.24 | [0.19, 0.28] | 0.33 | [0.29, 0.38] | 0.27 | [0.23, 0.31] |

Notes: Notes from Table 2 apply, with the exception that the model only has random intercepts but not a random age slope.

Appendix Table 19. Linear time trend coefficients for continuous score, models M1 and M2: Joint models for memory outcome and mortality

|  |  | **M1 - descriptive** | | **M2 - education** | |
| --- | --- | --- | --- | --- | --- |
|  | **N** | **b** | **95% CI** | **b** | **95% CI** |
| Trend coefficient |  |  |  |  |  |
| HRS | 188,526 | -0.02 | [-0.02, -0.01] | -0.07 | [-0.08, -0.06] |
| ELSA | 54,726 | 0.24 | [0.22, 0.26] | 0.15 | [0.13, 0.17] |
| SHARE | 190,493 | 0.27 | [0.26, 0.28] | 0.19 | [0.18, 0.20] |
|  |  |  |  |  |  |
| Association: Intercept |  |  |  |  |  |
| HRS |  | -0.17 | [-0.18, -0.16] | -0.16 | [-0.18, -0.15] |
| ELSA |  | -0.15 | [-0.19, -0.11] | -0.14 | [-0.18, -0.10] |
| SHARE |  | -0.15 | [-0.16, -0.14] | -0.16 | [-0.18, -0.15] |
|  |  |  |  |  |  |
| Association: Slope |  |  |  |  |  |
| HRS |  | -0.72 | [-0.77, -0.67] | -0.72 | [-0.77, -0.68] |
| ELSA |  | -0.58 | [-0.73, -0.44] | -0.57 | [-0.71, -0.43] |
| SHARE |  | -0.54 |  | -0.59 |  |
|  |  |  |  |  |  |
| Correlation b/w REs |  |  |  |  |  |
| HRS |  | -0.44 |  | -0.48 |  |
| ELSA |  | -0.32 |  | -0.39 |  |
| SHARE |  | -0.38 |  | -0.43 |  |

Notes: Estimates based on the joint modeling of the memory score and mortality. The longitudinal submodel is similar to Table 2, but age is modelled as continuous quadratic. The mortality submodel is based on a parametric proportional hazards model based on a Gompertz distribution. The two submodels are linked via random effects on the intercept and age slope, without any restrictions placed on their covariance matrix. The longitudinal submodel is directly comparable with Appendix Table 18, which constitutes an estimate of the submodel by itself. One can see from the first panel that the number of observations is smaller than in other tables since the time span format of the data required for duration analysis loses one observation per subject. The first panel also shows trend coefficients. The second and third panels show coefficient estimates for parameters that measure the impact of the random effects on the survival submodel. They are negative throughout, meaning that a random effect that increases the memory score is associated with a lower hazard of dying. The fourth panel depicts the estimated covariance between the two random effects. The number of failure events (deaths) is 24,028.

Appendix Table 20. Linear time trend coefficients for continuous score, models M1and M2: Joint models for memory outcome and attrition/mortality

|  |  | **M1 - descriptive** | | **M2 - education** | |
| --- | --- | --- | --- | --- | --- |
|  | **N** | **b** | **95% CI** | **b** | **95% CI** |
| Trend coefficient |  |  |  |  |  |
| HRS | 188,526 | -0.02 | [-0.03, -0.01] | -0.07 | [-0.08, -0.07] |
| ELSA | 54,726 | 0.24 | [0.22, 0.26] | 0.15 | [0.13, 0.17] |
| SHARE | 190,493 | 0.26 | [0.25, 0.27] | 0.19 | [0.18, 0.20] |
|  |  |  |  |  |  |
| Association: Intercept |  |  |  |  |  |
| HRS |  | -0.14 | [-0.15, -0.13] | -0.13 | [-0.14, -0.12] |
| ELSA |  | -0.15 | [-0.19, -0.11] | -0.14 | [-0.18, -0.10] |
| SHARE |  | -0.06 | [-0.07, -0.05] | -0.07 | [-0.08, -0.06] |
|  |  |  |  |  |  |
| Association: Slope |  |  |  |  |  |
| HRS |  | -0.63 | [-0.67, -0.59] | -0.63 | [-0.67, -0.58] |
| ELSA |  | -0.59 | [-0.73, -0.45] | -0.57 | [-0.71, -0.44] |
| SHARE |  | -0.44 |  | -0.46 |  |
|  |  |  |  |  |  |
| Correlation b/w REs |  |  |  |  |  |
| HRS |  | -0.45 |  | -0.49 |  |
| ELSA |  | -0.33 |  | -0.39 |  |
| SHARE |  | -0.40 |  | -0.44 |  |

Notes: Notes from Appendix Table 19 apply, except for a change in the failure event, which now combines mortality and attrition. Attrition is assumed for two contiguous observations that are non-responses and that occur at the end of a subject trajectory that does not end in death (i.e., the subject is censored). The number of failure events is 38,448, comprising of 24,028 deaths and 14,420 attritors.

# Appendix Figures

Appendix Figure 1. Time trend with confidence bounds for continuous score models M1

A: By Survey


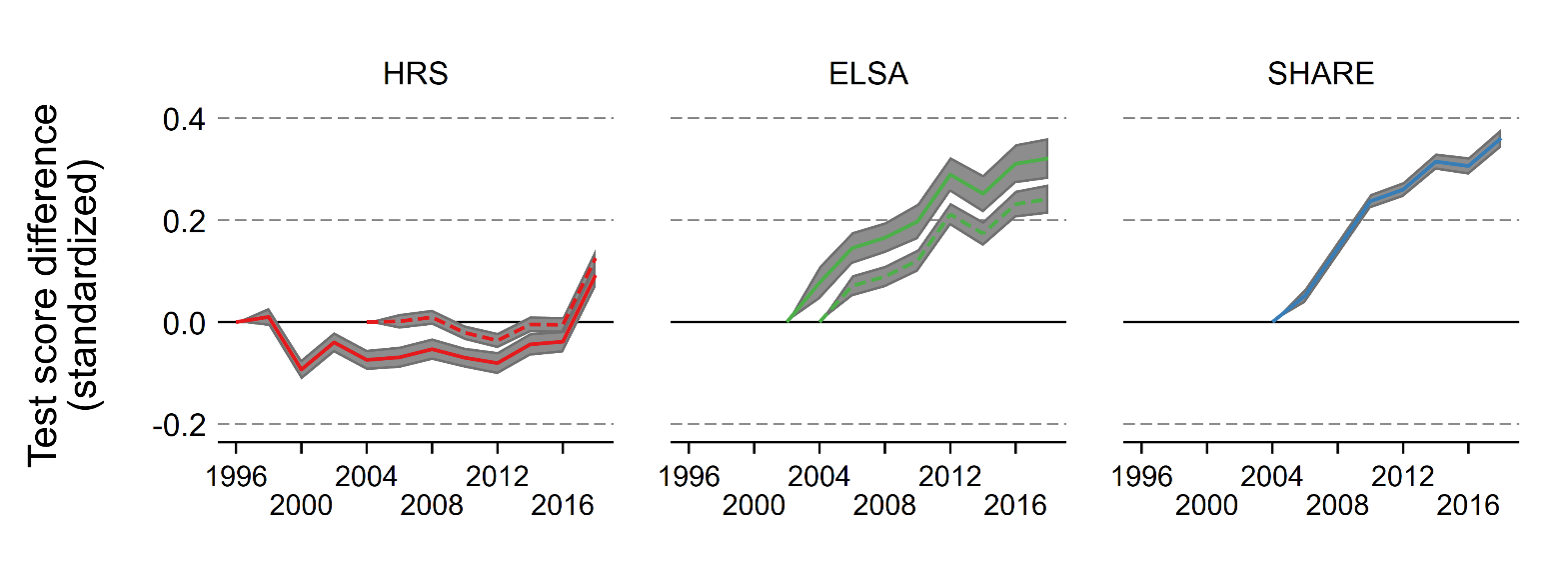


B: SHARE Total and Individual Countries


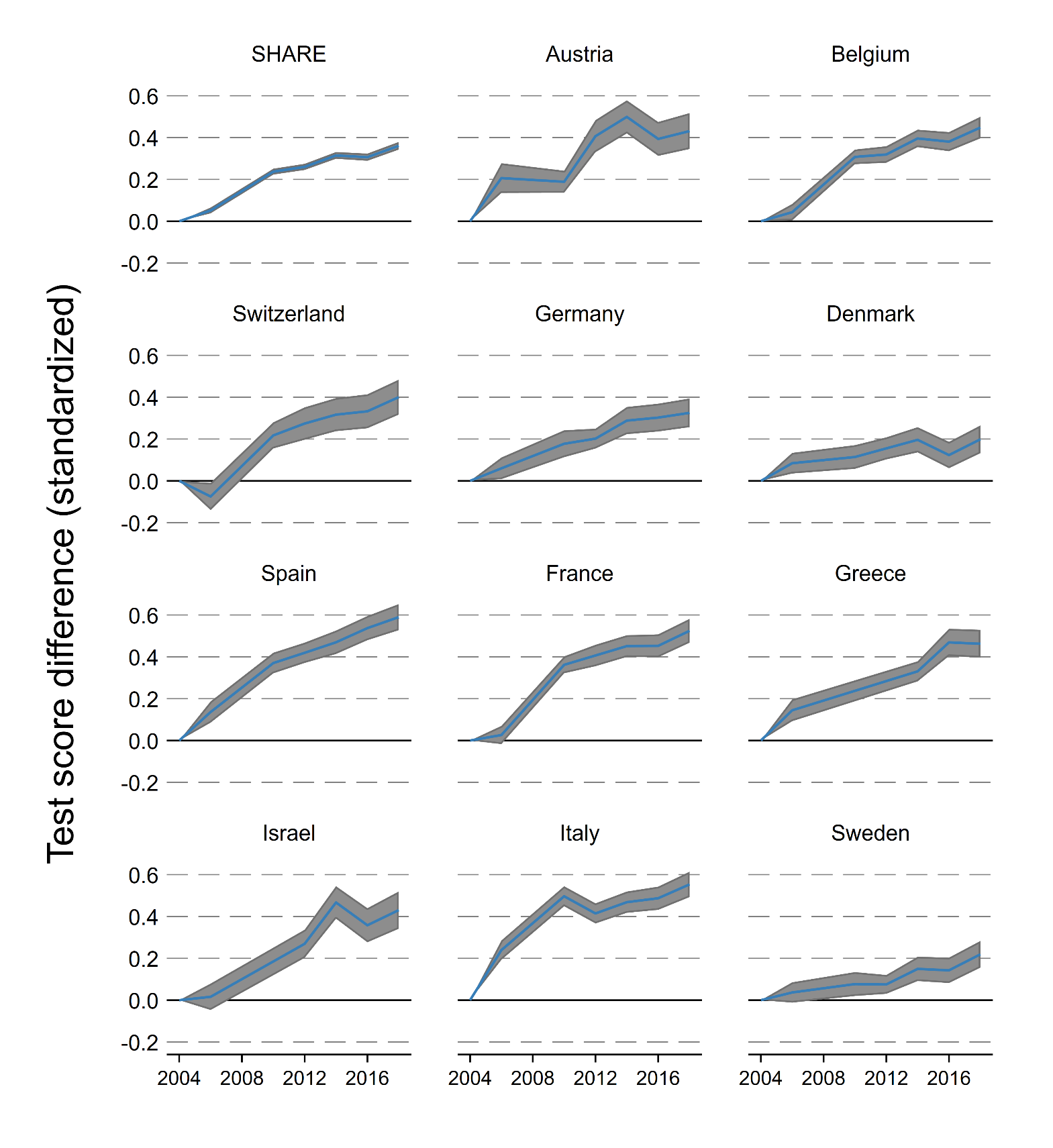


Notes: Coefficients for wave indicators and their confidence bounds for continuous score models M1. Notes from Figure 1 apply.

Appendix Figure 2. Change in binary memory impairment over 10 years, by subsamples of gender, education, and age group


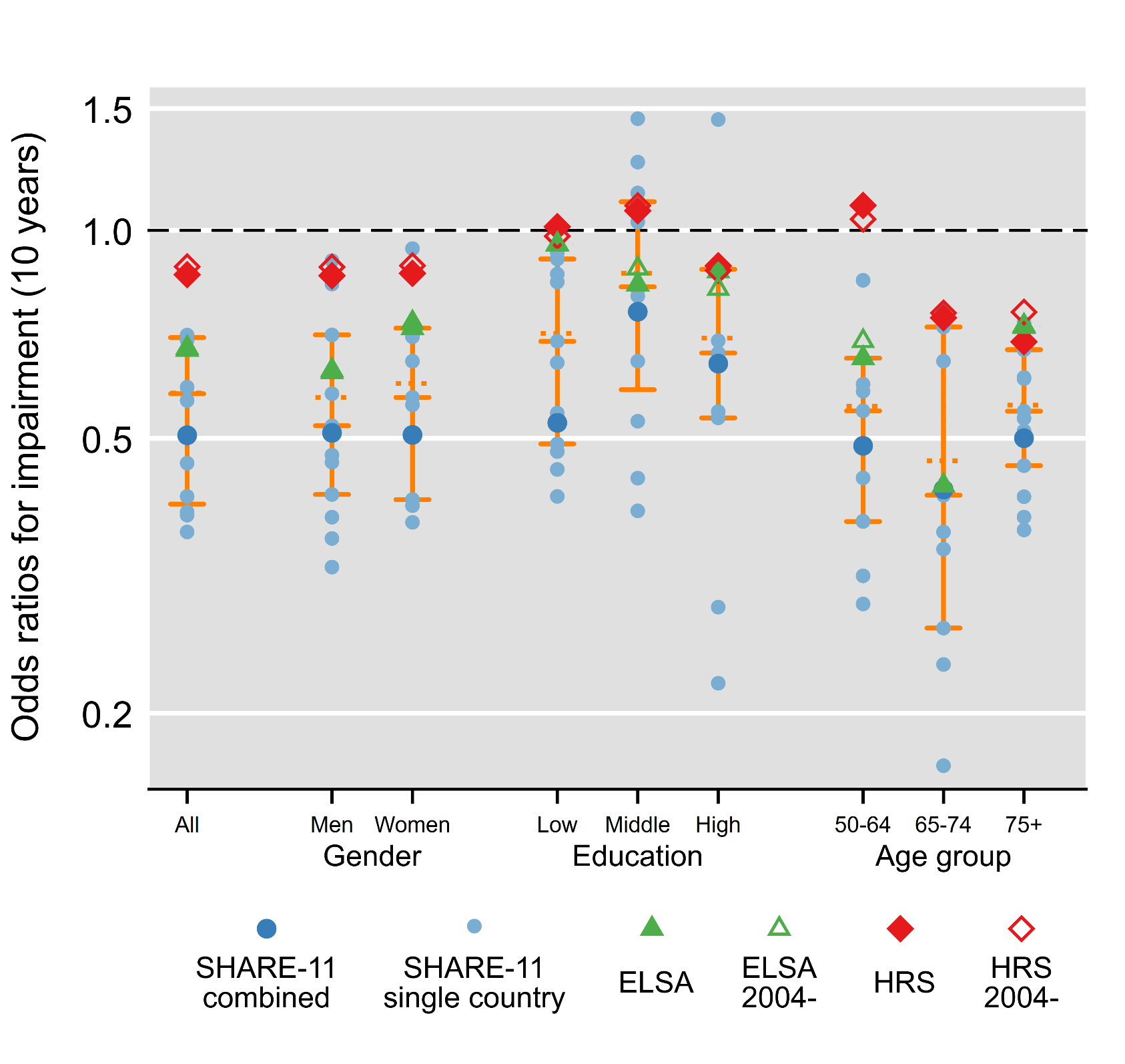


Notes: Based on survey/country-specific logistic regressions on binary memory impairment based on a country-specific cutoff value of 1.5 standard deviations below the country-specific mean for age group 50-69. Values shown are odds ratios on a log scale. 13 out of 144 coefficients (16 countries/samples, by 9 groups) could not be obtained due to model non-convergence and hence are omitted from the figure. Other than that, notes from Figure 2 apply.

Appendix Figure 3. Odds ratios for memory impairment of wave year indicator variables (model M1)


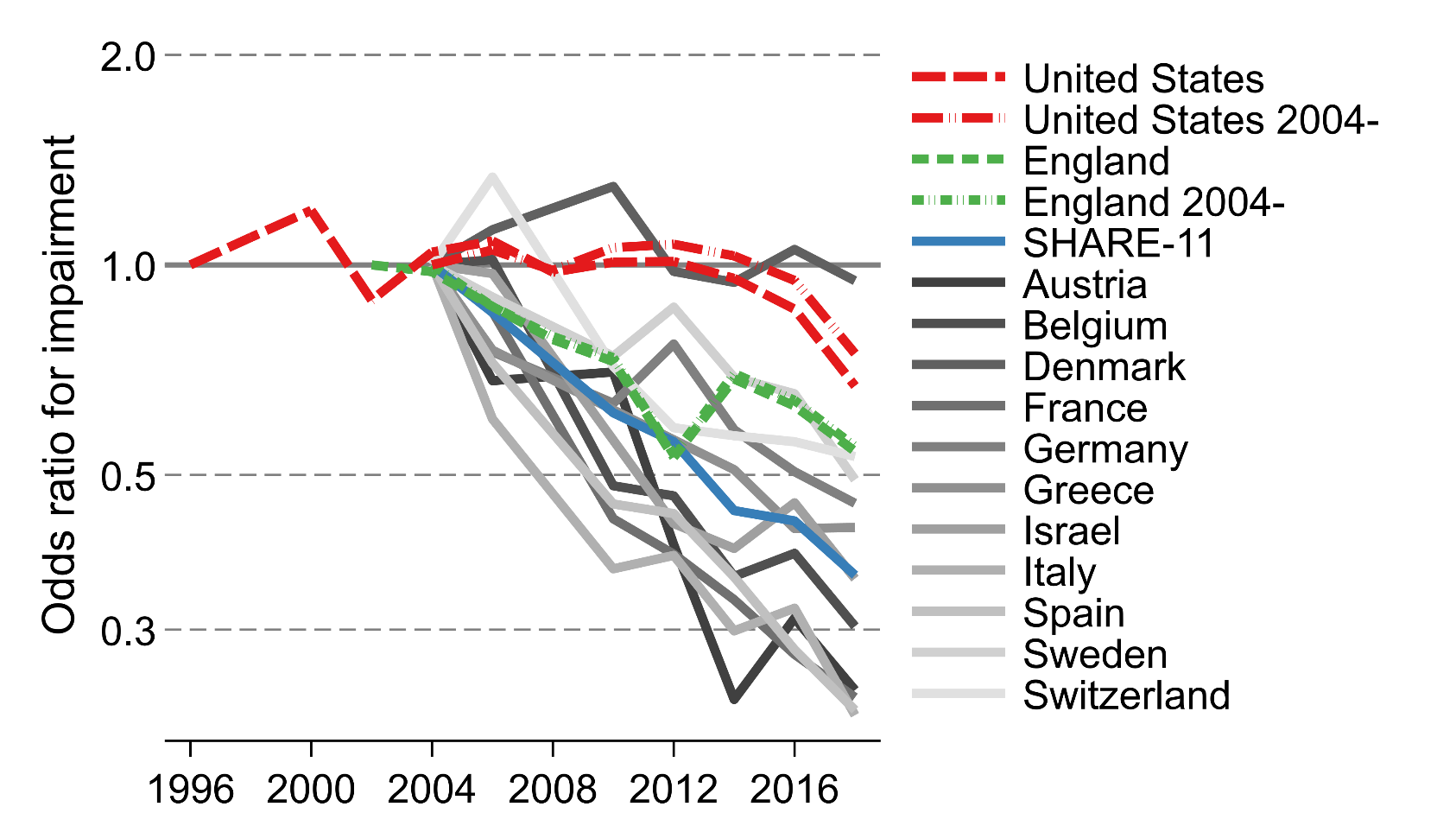


Notes: Based on survey/country-specific logistic regressions on binary impairment based on a country-specific cutoff value of 1.5 standard deviations below the country-specific mean for age group 50-69. Each line depicts odds ratios for impairment for different survey wave year indicator variables, relative to the first survey year in the sample (HRS: 1996; ELSA: 2002; SHARE: 2004). The underlying model M1 adjusts for age, gender, number of tests taken, and for the U.S., race/ethnicity and interview mode.

# References

1. Hale JM, Schneider DC, Gampe J, Mehta NK, Myrskylä M. Trends in the Risk of Cognitive Impairment in the United States. *Epidemiology*. 2020;31(5):745-754. doi:10.1097/EDE.0000000000001219
